# Disordered Gut Microbiota Correlates With Altered Fecal Bile Acid Metabolism and Post-cholecystectomy Diarrhea

**DOI:** 10.3389/fmicb.2022.800604

**Published:** 2022-02-18

**Authors:** Yayun Xu, Hui Jing, Jianfa Wang, Shilong Zhang, Qimeng Chang, Zhanming Li, Xubo Wu, Ziping Zhang

**Affiliations:** ^1^Department of Hepatopancreatobiliary Surgery, Minhang Hospital, Fudan University, Shanghai, China; ^2^Center for Traditional Chinese Medicine and Gut Microbiota, Minhang Hospital, Fudan University, Shanghai, China; ^3^Institute of Fudan-Minhang Academic Health System, Minhang Hospital, Fudan University, Shanghai, China; ^4^Department of Medical Oncology, Zhongshan Hospital, Fudan University, Shanghai, China

**Keywords:** post-cholecystectomy diarrhea, fecal bile acids, gut microbiota, 16S rRNA, UPLC/MS

## Abstract

Post-cholecystectomy diarrhea (PCD) is a common complication of gallbladder removal, and gut microbiota changes have been determined in PCD patients. Bile acid diarrhea (BAD) is supposed to be the main pathogenic factor for PCD due to the disrupted fecal bile acid metabolism in diarrheal patients. However, the profiling of bile acid metabolite alteration in PCD is unclear and whether changed gut microbiota and fecal bile acid metabolism are correlated is also underdetermined. The fecal bile acid metabolites from fecal samples were profiled by targeted UPLC/MS (ultra-high-performance liquid chromatography coupled with a triple-quadrupole mass spectrometer) and the composition of fecal bile acid metabolites in PCD patients was demonstrated to be distinct from those in Non-PCD and HC groups. In addition, the quantification of bile acid excretion in feces of diarrheal patients was significantly elevated. Furthermore, 16S rRNA sequencing results revealed that PCD patients had the lowest operational taxonomic units (OTU) and significant reduction in microbial richness and evenness. Bacterial composition was remarkably shifted in PCD patients, which mainly lay in dominated phyla *Firmicutes* and *Bacteroidota*. Besides, the co-abundance network among genus bacteria declined in PCD. Among the genera, *Prevotella*, *Enterococcus*, and *Erysipelotrichaceae_UCG-003* were enriched, but *Alistipes, Bacteroides*, *Ruminococcus*, and *Phascolarctobacterium* were reduced. Moreover, these disease-linked genera were closely associated with several diarrheal phenotypes. Notably, changed bile acid metabolites exhibited strong correlations with gut microbiota as well. Conclusively, this study reveals associations between PCD-linked microbes and bile acid metabolites, which may synergistically correlate to postoperative diarrhea.

## Introduction

Cholecystectomy remains the most common surgical procedure for symptomatic gallbladder diseases (Baron et al., 2015), and the mounting morbidity of cholelithiasis increases the performing frequency of this operation worldwide (Lammert et al., 2016). It has been considered as a safe surgical approach for a long time and accumulating advances in minimally invasive surgery promote its clinical application (Baron et al., 2015). However, increased risks of many complications in patients who underwent cholecystectomy are obtaining growing attention (Ioannou, 2010; Chen et al., 2014), among which post-cholecystectomy diarrhea (PCD) highly occurred (Fort et al., 1996; Damsgaard et al., 2018; Farrugia et al., 2021). Postoperative diarrhea, characterized by persistent altered bowel habits including chronic diarrhea, defecation urgency, and increased defecation frequency, reduces the quality of life greatly in a huge number of outpatients (Lamberts et al., 2012). Recently, the incidence of PCD has been reported to increase from 2.1 to 57.2%; however, the mechanisms responsible for the persistence of PCD are still unknown (Fort et al., 1996; Damsgaard et al., 2018; Farrugia et al., 2021).

Bile acid diarrhea (BAD) is recognized as the most common etiology for postoperative diarrhea on account of the disordered bile acid metabolism in feces of PCD patients by SeHCAT scanning test (Sciarretta et al., 1992). Gallbladder plays a vital role in storing and concentrating bile acids during interdigestive period, but ablating these functions accelerates the enterohepatic circulation of bile acids and eventually increases the fecal bile acid concentration in colon (Housset et al., 2016). BAD is also called bile acid malabsorption because the diarrhea is caused by the body being unable to restrain losing water and salts into the bowel (Camilleri and Vijayvargiya, 2020). Compared to patients with irritable bowel syndrome with diarrhea (IBS-D), BAD patients feature uniquely in bile acid metabolism, for example, elevated cholic acid and chenodeoxycholic acid (Sagar et al., 2020). However, profiling results of fecal bile acid metabolism in PCD patients are still unclear.

Human gastrointestinal tract resides immense gut microbiota, which outnumbers host cells approximately 10-fold (Sommer and Backhed, 2013). Mounting lines of evidence have elucidated the pivotal roles of gut microbiota and their metabolic derivatives in maintaining normal physiologies (Sommer and Backhed, 2013; Schroeder and Backhed, 2016). Besides, changed gut microbiota has been verified as a contributing factor for many gastrointestinal diseases, for example, IBS-D (Zhao et al., 2020) and inflammatory bowel disease (IBD) (Vich et al., 2018). Negative association between fecal consistency and microbial richness implies the fundamental part of gut microbiota in the pathogenesis of diarrhea (Vandeputte et al., 2016). In addition, gut microbiota is significantly altered after cholecystectomy (Yoon et al., 2019), and this has been evidenced in a self-contrast research by comparing pre- with postoperative state as well (Keren et al., 2015). Furthermore, a recent study reports that disturbed gut microbiota is observed among patients with PCD (Li et al., 2021). Bile acids directly influence bacterial growth by disrupting bacterial cell wall architecture or synthesizing antimicrobial peptide cathelicidin (D’Aldebert et al., 2009; Wahlstrom et al., 2016). Since cholecystectomy augments the bile acid flow into colon and induces significant alteration on bile acid metabolism in mice (Zhang et al., 2017), whether changes in gut microbiota in diarrheal patients is correlated to the altered bile acid metabolites remains unknown.

To verify this, we quantified fecal bile acid metabolites and microbial composition changes in individuals, namely, PCD, Non-PCD, and healthy controls. Our results indicated distinct fecal bile acid metabolism in the PCD group, which was accompanied with imbalanced status of gut microbiota. More importantly, altered gut microbiota and metabolites were strongly associated. Herein, our findings firstly revealed the characteristics of bile acid metabolism of post-cholecystectomy and the tight correlations among metabolism of bile acids, gut microbiota, and PCD, which provided novel clues for the pathogenesis, diagnosis, and treatment of PCD.

## Materials and Methods

### Study Design

Outpatients who underwent cholecystectomy from January 2019 to December 2019 in Minhang Hospital, Fudan University for gallbladder disease were followed up by telephone. According to the guideline for the investigation of chronic diarrhea in adults, British Society of Gastroenterology, 3rd (Arasaradnam et al., 2018), those who complained of loose stool, increased defecation frequency, or urgency after cholecystectomy were considered as PCD. A total of 48 eligible outpatients were included and were divided into a PCD group (*n* = 23) and a Non-PCD group (*n* = 25). In addition, 22 healthy individuals were collected and used as the control (HC) group (*n* = 22). Clinical characteristics including BMI, defecation frequency per day, stool output, and fecal consistency by Bristol stool score (BSS) were recorded and are shown in Table 1. Morning first stool samples were obtained from donors and suspended in an equal volume (w/v) PBS containing 20% glycerin and then frozen in liquid nitrogen immediately and preserved in −80°C until use. Bile acid metabolites and gut microbiota were measured by targeted ultra-high-performance liquid chromatography coupled with a triple-quadrupole mass spectrometer (UPLC/MS) and 16S rRNA gene sequencing, respectively.

**TABLE 1:** Demographic information of healthy controls and non-diarrheal and diarrheal patients after cholecystectomy in this study.

	HC	Non-PCD	PCD
Cases	22	25	23
Sex			
Male	10	13	12
Female	12	12	11
Age	40.01 ± 2.51	47.04 ± 2.58	42.96 ± 2.11
Weight (kg)	65.00 ± 1.90	66.66 ± 1.60	69.09 ± 1.85
Height (m)	1.70 ± 0.02	1.66 ± 0.01	1.67 ± 0.01
BMI (kg/m^2^)	22.57 ± 0.44	24.20 ± 0.50	24.89 ± 0.62
Cholecystolithiasis	0	25	23
Cholecystectomy	0	25	23
Defecatory frequency (/day)	1.07 ± 0.04	1.22 ± 0.05	3.04 ± 0.20^a,b^
Stool output (g/day)	117.17 ± 5.84	129.80 ± 11.94	342.09 ± 11.16^a,b^
Bristol stool score	2.73 ± 0.21	2.92 ± 0.25	5.61 ± 0.19^a,b^
Total	*n* = 70		

*BMI, body mass index, HC, healthy control, PCD, post-cholecystectomy diarrhea, Non-PCD, non-PCD. Data are shown as mean ± standard error mean (SEM). Differences between two groups of each bile acid were compared by Mann–Whitney U-test.*

*^a^p < 0.05 compared with the Non-PCD group and ^b^p < 0.05 compared with HC.*

### Participants Recruiting and Sampling

PCD and Non-PCD patients were recruited *via* telephone. Patients were selected according to the following criteria: (1) underwent cholecystectomy in 2019; (2) within 18–65 years old; (3) provided written informed consent; and (4) PCD patients were selected when they met the diarrhea criteria, such as loose stool, increased defecation frequency (at least 3 times per day) or urgency, and significant altered fecal appearance graded by BSS. Patients were excluded if they have (1) surgical histories of gastrointestinal tract; (2) a medical history of irritable bowel syndrome (IBS), inflammatory bowel disease (IBD), constipation, and infective or idiopathic diarrhea; (3) a medication history of antibiotics, probiotics, or medicines known to affect gut microbiota; and (4) a history of severe chronic diseases.

Healthy controls (HC) were recruited voluntarily through our advertisements by the following criteria: (1) age within 18–65 years old; (2) provided written informed consent; (3) no gallbladder removal surgery or other history of gastrointestinal surgery; (4) fecal consistency scores of 3 and 4 by BSS; (5) no antibiotics or probiotics administration.

### Ethics Approval

This study was authorized by the ethics committee of Minhang Hospital, Fudan University and written informed consent was obtained from all volunteers.

### Targeted Bile Acid Metabolism in Fecal Samples

Fecal bile acid metabolites from 43 individuals (Supplementary Table 1) were analyzed by UPLC/MS (Waters Acquity UPLC, AB SCIEX 5500 QQQ-MS) as previously reported (Zhao et al., 2020). The collected samples were dried in a lyophilizer. Then, 50 mg of each sample was mixed with 800 μl of precooled methanol containing 20 ng/ml internal standards (Shanghai yuanye Bio-Technology Co., Ltd., China), vortexed for 1 min, and incubated at 4°C for 30 min. After centrifugation at 12,000 rpm for 10 min, the supernatants were diluted 100 times with methanol (containing 20 ng/ml internal standard) and quantified in multiple reaction monitoring (MRM) mode. The acquisition data were analyzed by MultiQuant software (AB sciex, United States) and the concentration of individual bile acid was calculated by comparing with internal standard. Total fecal bile acid excretion of each donor was calculated by the concentration (ng/g) × defecation output (g).

### Extraction of Microbial Genome DNA and 16S rRNA Amplicon Sequencing

According to the manufacturer’s instructions, total genome DNA from human fecal samples were extracted by a DNA extraction kit (TIANGEN, China). In the template of microbial DNA, hypervariable regions (V3–V4) of the 16S rRNA gene were amplified using specific bacterial primers (338 F and 806 R) with the barcode and processed to sequencing libraries through TruSeq ^®^ DNA PCR-Free Sample Preparation Kit (Illumina, United States). The forward primer was 5′-ACTCCTACGGGAGGCAGCAG-3′ and the reverse primer was 5′-GGACTACHVGGGTWTCTAAT-3′. Sample amplicons were sequenced on an Illumina HiSeq platform (Illumina, MiSeq, United States) and 250-bp paired-end reads were generated. The entire 16S rRNA database was deposited to the public platform of NCBI short-read archive with BioProject number PRJNA777105.

### Data Bioinformatic Processing

After splitting, assembly, filtration, and chimera removal, sequences with 97% or more similarity were assigned to the same operational taxonomic units (OTU). Alpha-diversity was applied to analyze complexity of gut microbiota diversity and were calculated with QIIME (Version 1.9.1). Beta-diversity was used to evaluate differences among samples, which was analyzed by Bray–Curtis dissimilarity and Jaccard similarity index. Bacterial community distance was estimated using non-metric multidimensional scaling (NMDS) assay by vegan package, principal coordinate analysis (PCoA) was performed by ggplot2 package, and unweighted pair−group method with arithmetic means (UPGMA) analysis was carried out by R phangorn package among samples. The co-occurrence network was applied to analyze associations between altered genus bacteria and other genera by Spearman correlation based on their relative abundance data (Zhang et al., 2021). Multiplicity testing was conducted by Benjamini, Hochberg, and Yekutieli false discovery rate (FDR), and *p*_fdr_ < 0.05 was considered significant after adjustment. Significant correlations (*p*_fdr_ < 0.05, ∣*r*∣ > 0.6) were visualized by Cytoscape (v3.8.2) and parameters of co-occurrence network are shown in Supplementary Table 2. Similarly, the integrative correlation network of specific microbial taxa with diarrheal phenotype was analyzed using the same method. Receiver operating characteristic (ROC) curve was applied to distinguish PCD from Non-PCD. The bile acid data were displayed in Orthogonal Projections to Latent Structures Discriminant Analysis (OPLS-DA, ropls package) and heatmap plot (pheatmap package). The associations between gut microbes and bile acid metabolites were analyzed by Spearman correlation (PResiduals package).

### Statistics

Data were expressed as the mean ± standard error of the mean (SEM). GraphPad Prism 8.0 (GraphPad Software, Inc., San Diego, CA, United States) and R software (version 3.6.3) were applied for all statistical analysis. Unpaired non-parametric Mann–Whitney test was conducted for difference comparison. Multiplicity testing was conducted, and *p*-value was adjusted using the methods of Benjamini, Hochberg, and Yekutieli to control the FDR. *p*_fdr_ < 0.05 was considered significant.

## Results

### The Baseline Characteristics of Eligible Patients

**FIGURE 1:**
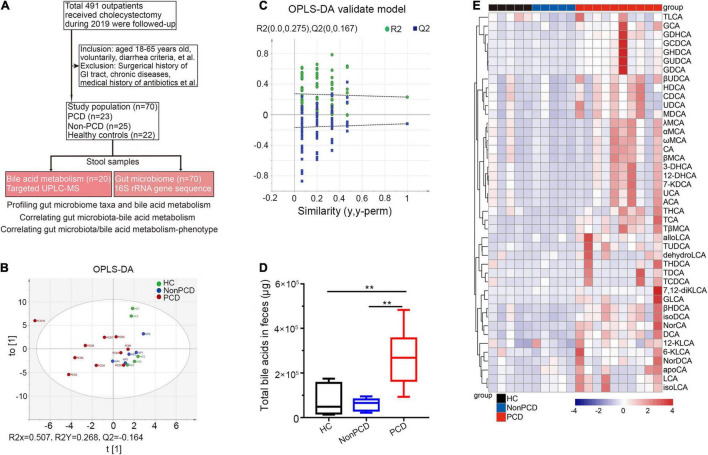
Metabolic changes of fecal bile acids in PCD patients compared to Non-PCD and HC. **(A)** Flow chart of this study. **(B)** Orthogonal Projections to Latent Structures Discriminant Analysis (OPLS-DA) plot revealing metabolic deviation of fecal bile acids composition from PCD (*n* = 10) to Non-PCD (*n* = 5) patients and HC (*n* = 5) (R2Y = 0.268, *Q*2 = -0.164). **(C)** Representative permutations plot was applied as OPLS-DA validate model among three groups in bile acid metabolism. **(D)** Box plot presenting excessive total bile acids output in feces from PCD, compared to Non-PCD and HC. Data are shown as Min to Max; *n* = 5 for Non-PCD and HC, 10 for PCD patients **p < 0.01. **(E)** Representative heatmap showing quite distinct fecal content of these 43 bile acids detected among three groups; total fecal bile acid excretion of donor was calculated by the concentration (ng/g) × defecation output (g).

A total of 105 (21.38%) patients complained of altered bowel habits after cholecystectomy. In this study, we recruited 70 donors, including 23 PCD patients, 25 Non-PCD patients, and 22 HC, and collected their fecal samples, as shown in the flow chart (Figure 1A). Overall, the baseline characteristics including age, sex, height, and BMI were comparable across three groups. PCD patients exhibited increased defecation output and frequency, and higher BSS, which met the diagnostic criteria for diarrhea (Table 1). Microbial genome DNA were extracted from fecal samples and sequenced by 16S rRNA sequencing analysis and targeted metabolic profiling on fecal bile acids was performed on a subset of 20 samples (Figure 1A).

### Considerable Alterations in Bile Acid Metabolic Status Were Observed Among Post-cholecystectomy Diarrhea Patients

Next, we profiled a panel of 43 fecal bile acid metabolites (Supplementary Table 1) by targeted UPLC/MS. First, the OPLS-DA indicated distinct clustering pattern across three groups in bile acid composition (Figure 1B), and the goodness of fit and high prediction (R2Y = 0.268, *Q2* = -0.164) of this model showed good performance (Figure 1C). This demonstrated a significant discrimination of bile acid structure in diarrheal patients. PCD patients displayed significantly increased concentration (Supplementary Figure 1A) and total bile acid content in feces, compared to either Non-PCD patients or HC (Figure 1D). More specific, a panel of bile acids was displayed and we found a remarkable difference in fecal bile concentration (Supplementary Figure 1B) and content (Figure 1E) in PCD patients, in contrast to either Non-PCD or HC. Among them, some primary conjugated bile acids, such as taurocholic acid (TCA) and tauro β-muricholic acid (TβMCA), and some secondary bile acids, such as tauroursodeoxycholic acid (TUDCA) and deoxycholic acid (DCA), were greatly abundant (Figure 1E and Supplementary Figure 1B). Collectively, patients with PCD had a distinct alteration in fecal bile acid metabolism.

### Decreased Diversity and Disturbed Composition of Gut Microbiota in Post-cholecystectomy Diarrhea Patients

**FIGURE 2:**
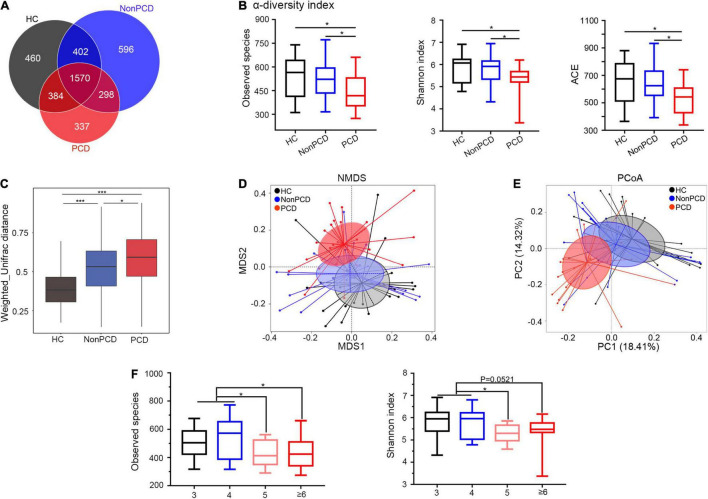
Attenuated alpha diversity and altered gut microbiota composition in patients with PCD. **(A)** Overlapping Venn diagram showing the operational taxonomic units (OTU) of microbial taxa among these three groups. **(B)** Decreased alpha-diversity of gut microbiota in the PCD group by observed species, Shannon index and ACE. **(C)** Box plot for beta-diversity index in three groups based on weighted Unifrac distance (Wilcox rank sum test). **(D)** The microbial distributions among three groups were indicated by non-metric multidimensional scaling (NMDS) plot with cluster. **(E)** Differences of bacterial taxa were analyzed by principal coordinate analysis (PCoA) among these groups. **(F)** Observed species and Shannon index distributed in four classes based on BSS. Data are expressed in box plot as Min to Max; *n* = 22 for HC, 25 for Non-PCD, and 23 for PCD patients. *p < 0.05 ***p < 0.005. BSS, Bristol stool score.

Due to anatomic and dynamic reasons, the unabsorbed bile acids have a great impact to the microbial community, so sequencing flowing bile acids to intestine needs to be emphasized (Joyce and Gahan, 2016). To investigate gut microbiota changes among the three groups, 16S rRNA gene sequencing was conducted and microbial DNA was assigned to OTU. The sequencing depth of 16S rRNA sequencing was reflected in rarefaction curves, and reduced microbial richness and evenness in PCD patients was intuitively shown by rank abundance curve (Supplementary Figures 2A,B). Overlapping OTU among the three groups were shown by the Venn diagram (VennDiagram package) in Figure 2A. A total of 1,570 OTU were shared mutually, but 337 OTU were unique in the PCD group, evidently fewer than HC and Non-PCD groups. Community richness and evenness of PCD group declined significantly as indexed by bacterial alpha-diversity, in contrast to HC and Non-PCD groups (Figure 2B and Supplementary Figure 2C). Besides, the variability of bacterial structure among groups was analyzed and beta distance by Weighted Unifrac in PCD patients was significantly higher than that in HC and Non-PCD patients (Figure 2C). To pinpoint microbial alteration, the NMDS model was implemented. As shown in Figure 2D, the PCD group cluster separated distinguishingly from HC and Non-PCD groups, and the significance between PCD and Non-PCD was achieved by ANOSIM analysis (*R* = 0.195, *p* = 0.001, R vegan package, Supplementary Figure 2D). Furthermore, PCoA by Bray–Curtis distance indicated an obvious variability in gut microbiota composition among three groups (Figure 2E). This was also confirmed by unweighted pair-group method with arithmetic means (UPGMA) on binary-Jaccard distance analysis (Supplementary Figure 2E). These results demonstrated dramatically reduced gut microbiota richness and tremendous changes in bacterial composition in PCD patients. Besides, observed species and Shannon index were significantly lower in Bristol Stool Scale (BSS) 5 and ≥ 6 groups, in contrast to BSS 3 and 4 (Figure 2F) in our study, which was consistent with previous results (Vandeputte et al., 2016; Asnicar et al., 2021).

### Identification of Altered Bacterial Taxa in Feces From Individuals With Post-cholecystectomy Diarrhea

**FIGURE 3:**
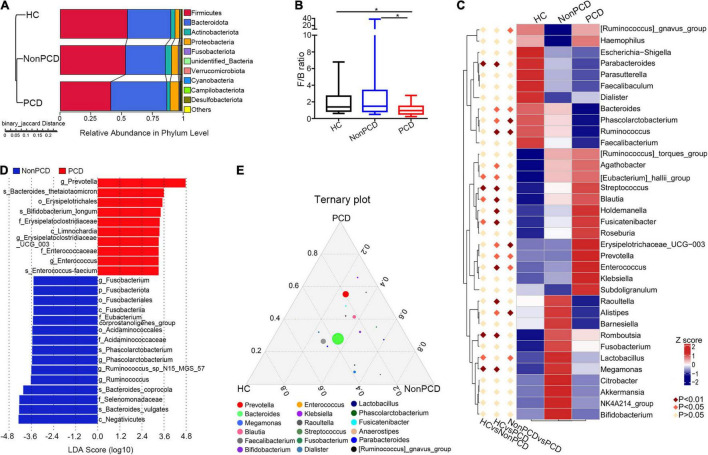
Identification of altered bacterial taxa involved in PCD patients. **(A)** Grouped unweighted pair-group method with arithmetic means (UPGMA) tree based on binary-Jaccard distance showing microbial similarity among groups (left panel) and relative abundance of top 10 bacteria in phylum level (right panel). **(B)** Decreased *Firmicutes*/*Bacteroidota* ratio in the PCD group, compared to Non-PCD and HC groups. Data are presented in box plot as Min to Max; *n* = 22 for HC, 25 for Non-PCD, and 23 for PCD patients. *p < 0.05. **(C)** Representative heatmap analyzed by MetaStat method showing microbial differences in genus level among three groups. **(D)** Linear discriminant analysis (LDA) effect size (LEfSe) was applied to identify differential abundance of genera in Non-PCD and PCD groups (bacteria whose LDA score > 3.0 were plotted). **(E)** Ternary plot showing differential microbial genera in these groups; the three vertexes of this graph were denoted HC, Non-PCD, and PCD, respectively. Different dots represent corresponding genera and the size for relative abundance of microbe. The closer to vertexes, the greater contribution to groups.

A total of 37 microbial taxa were identified between Non-PCD and PCD patients by *t*-test (Supplementary Table 3), namely, 2 phyla, 3 classes, 8 orders, 9 families, and 15 genera (mean relative abundance > 0.1%, *p* < 0.05). At phylum, *Firmicutes* was under-represented in the PCD group, and *Bacteroidota* and *Proteobacteria* were over-represented (Figure 3A). Besides, a reduced *Firmicutes*/*Bacteroidota* ratio of PCD patients indicated a conspicuous change in phylum (Figure 3B). In genus level, the top 30 fecal bacteria (Supplementary Figure 3A) were further analyzed using Metastats and 9 crucial microbes were exclusively identified in PCD, namely, elevated *Prevotella*, *Enterococcus*, *[Ruminococcus]_gnavus_group*, and *Erysipelotrichaceae_UCG-003*, and reduced *Alistipes*, *Lactobacillus*, *Ruminococcus*, *Phascolarctobacterium*, and *Bacteroides* (Figure 3C).

To rank the greatest differences of abundant genera between Non-PCD and PCD groups, the linear discriminant analysis effect size (LEfSe) analysis (LEfSe software, version 1.0) was conducted. The cladogram indicated significant microbial alteration in Non-PCD and PCD patients and highlighted 15 key genus microbes (Supplementary Figure 3B). According to LDA score (LDA > 3.0), the greatest difference of each bacterium was exhibited in Figure 3D. A total of 10 genus, including *Prevotella*, *Enterococcus*, and *Erysipelotrichaceae_UCG-003*, were enriched in PCD, while 15 microbes, including *Ruminococcus*, *Phascolarctobacterium*, and *Fusobacterium*, were enriched in Non-PCD. Furthermore, a ternary plot model (R vcd package) was generated to pinpoint specific genus bacteria responsible for the microbial difference. As shown in Figure 3E, *Prevotella*, *Klebsiella*, and *Streptococcus* were located near the PCD group; in contrast, *Raoultella*, *Megamonas*, *Lactobacillus*, and *Fusobacterium* leaned toward the Non-PCD group.

### Correlation of Gut Microbiota With Diarrheal Clinical Phenotypes

**FIGURE 4:**
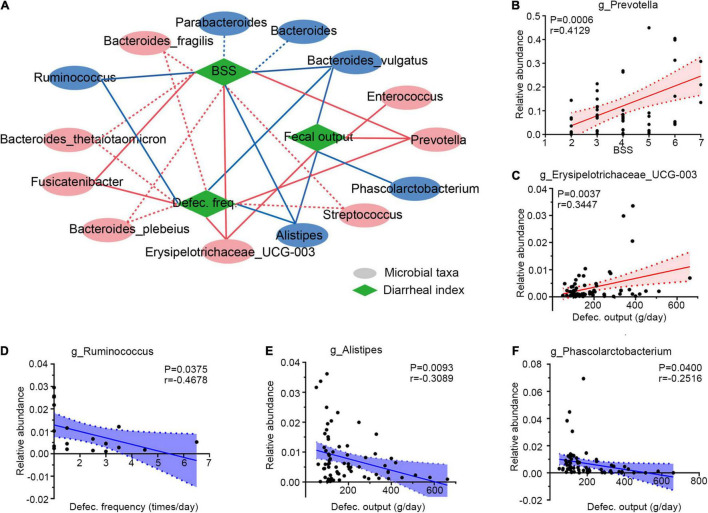
Integrative correlation network of specific and diarrheal phenotype. **(A)** Correlation network displaying significant associations between altered gut microbes with diarrheal parameters. Microbes and clinical indexes were denoted by circles and rhombus; red color shows an increase (positive related) and blue shows a decrease (negative related) in diarrheal individuals. Any correlations with *p* > 0.05 and ∣*r*∣ < 0.25 were abolished; dotted and solid lines were significant and suggestive associations. **(B–F)** Representatives of special genera-clinical indexes linear correlations. Associations between *Prevotella* and BSS (B), *Erysipelotrichaceae_UCG-003* and defection output **(C)**, *Ruminococcus* and defection frequency (D), *Alistipes* and defecation output **(E)**, and *Phascolarctobacterium* and defecation output **(F)**. Dotted lines show error bar, and areas between two dotted lines denote 95% confidence interval; red indicates positive correlations and blue denotes negative correlations. Single datum point was abandoned once it deviated the correlation line dramatically. BSS, Bristol stool score, defec., defection.

Then, Spearman correlation analysis was applied to determine the associations between gut microbiota and diarrheal clinical indexes (defecation frequency, BSS, and defecation output). Six genera were selected for their significant correlations with diarrheal phenotypes, as illustrated in Figure 4A. These altered microbes in PCD patients displayed strong associations with diarrheal clinical parameters. For example, overabundant *Prevotella* (Figure 4B and Supplementary Figure 4A), as well as *Erysipelotrichaceae_UCG-003* (Figure 4C and Supplementary Figure 4B), *Enterococcus* (Supplementary Figure 4C), and *Fusicatenibacter* (Supplementary Figure 4D) were positively associated with these clinical phenotypes. However, *Ruminococcus* (Figure 4D and Supplementary Figure 4E), *Alistipes* (Figure 4E and Supplementary Figure 4F), and *Phascolarctobacterium* (Figure 4F) presented negative correlations.

### Decreased Co-occurrence Network Among Bacteria in Genus of Post-cholecystectomy Diarrhea Patients

**FIGURE 5:**
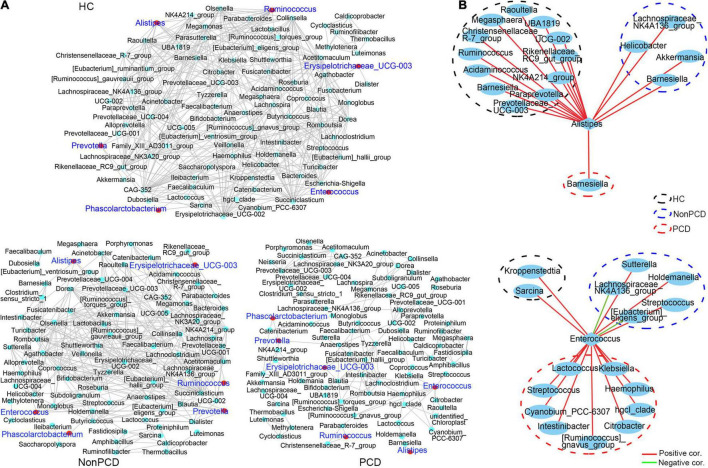
Decreased co-occurrence network among genus bacteria in PCD patients. **(A)** Representative co-abundance network of genera in three groups, respectively. Based on the database of abundance of each taxon in genus, correlations among top 100 genera were calculated and correlation coefficients (*p*_fdr_ < 0.05, ∣*r*∣ > 0.6) were regarded as clear links and visualized by Cytoscape v3.8.2. False discovery rate (FDR) was controlled using the methods of Benjamini, Hochberg, and Yekutieli after the multiplicity testing and adjusted *p*_fdr_ < 0.05 was considered significant. Green dots represent genus bacteria and the gray line between two dots represents mutual correlation; red dots were labeled blue for 6 key diarrhea-related taxa in three groups. Gut microbiota in HC and Non-PCD groups are closely connected, but tended to assemble to several modules separately in PCD. **(B)** Reduced co-abundance network of *Alistipes* (upper panel) and augmented co-abundance network of *Enterococcus* (lower panel) with other genus microbes in PCD. Blue circles represent bacteria in genus, red lines denote positive correlations, and green lines indicate negative correlations.

Gut microbiota plays an important role in maintaining the health of bacterial microenvironment and equates to bacterial diversity (Yang et al., 2017), and the co-occurrence network model is widely used in microbial interaction analysis (Qin et al., 2012; Jeffery et al., 2020). Hence, a co-occurrence network to decipher associations among genus bacteria was constructed using Spearman correlation, and significant correlations (*p*_fdr_ < 0.05, ∣*r*∣ > 0.6) were selected and visualized. Generally, there was a reduced network density in PCD with 166 correlations, but 325 correlations in Non-PCD and 403 in HC (Figure 5A and Supplementary Table 4), which was confirmed by a lower graph density value (Supplementary Table 2). Besides, we found more co-abundance correlations (89.2%) in the PCD group than the other groups (Supplementary Table 4). Intriguingly, bacteria in PCD patients tended to assemble to several modules separated from each other, but connected more closely in HC and Non-PCD (Figure 4A), and it was verified by higher modularity in the PCD group (Supplementary Table 2). Furthermore, the correlations between these key diarrhea-linked bacteria and others were built as well. *Enterococcus* augmented its associations with others in PCD patients (Figure 5B, upper panel), but *Alistipes* reduced its associations in PCD (Figure 5B, lower panel), compared to HC and Non-PCD. Besides, *Prevotella* (Supplementary Figure 5A), *Fusicatenibacter* (Supplementary Figure 5B), *Ruminococcus* (Supplementary Figure 5C), *Phascolarctobacterium* (Supplementary Figure 5D), and *Bacteroides* (Supplementary Figure 5E) exhibited decreased co-occurrences with other genera in PCD patients as well.

### Correlation Between Specific Gut Microbiota and Bile Acid Metabolites

**FIGURE 6:**
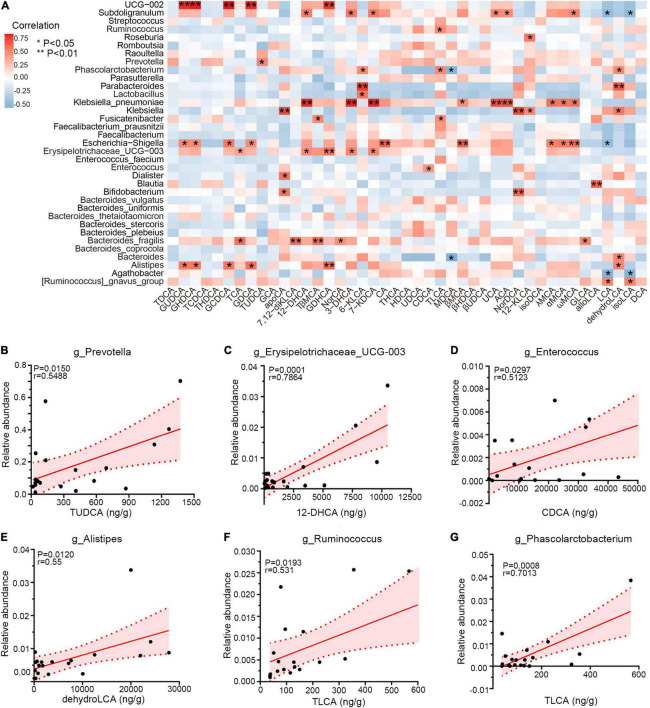
Correlations among gut microbiota and bile acid metabolites. **(A)** Heatmap of Spearman correlation between relative abundance of gut microbiota and exact concentration of fecal bile metabolites (*n* = 20), *p < 0.05, **p < 0.01. **(B–G)** Representative linear correlations of diarrhea-linked genera and bile metabolites. Association between *Prevotella* and TUDCA (B), *Erysipelotrichaceae_UCG-003* and 12-DHCA **(C)**, *Enterococcus* and CDCA **(D)**, *Alistipes* and dehydroLCA **(E)**, *Ruminococcus* and TLCA **(F)**, and *Phascolarctobacterium* and TLCA **(G)**. Pink dotted lines show error bar and pink area for 95% confidence interval; red denotes positive association. Single datum point was abandoned once it deviated the correlation line dramatically.

Emerging perspectives on liver–bile acid–gut microbiota axis have emphasized the inter-crosstalk between bile acids and intestinal bacteria in regulating gastrointestinal health (Jia et al., 2018). To investigate the associations between genera and bile acids among three groups, Spearman correlation was performed. As shown in Figure 6A, PCD-linked bacteria were positively correlated with bile metabolites generally. Notably, *Prevotella* and TUDCA, enriched simultaneously in PCD patients, were positively associated (*r* = 0.55, *p* = 0.015, Figure 6B), and overabundant *Erysipelotrichaceae_UCG-003* exhibited positive correlation with 12-dehydrocholic acid (12-DHCA) (*r* = 0.79, *p* = 0.0001, Figure 6C). Additionally, *Enterococcus* was positively associated with chenodeoxycholic acid (CDCA) (*r* = 0.51, *p* = 0.0297, Figure 6D), and the positive associations were also presented between *Alistipes* with dehydrolithocholic acid (dehydroLCA) (*r* = 0.55, *p* = 0.0120, Figure 6E), *Ruminococcus* with taurolithocholic acid (TLCA) (*r* = 0.53, *p* = 0.0193, Figure 6F), and *Phascolarctobacterium* with TLCA (*r* = 0.70, *p* = 0.0008, Figure 6G), respectively.

**FIGURE 7:**
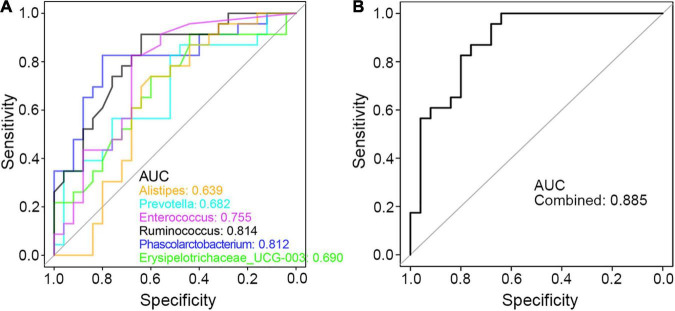
ROC curve in discriminating PCD and Non-PCD by diarrhea-related genera. Area under curve (AUC) of single **(A)** and combined **(B)** genus taxon, respectively, in discriminating PCD and Non-PCD.

Finally, to seek the potential utility of these genera in clinical diagnosis of PCD, ROC curve was applied and the area under the curve (AUC) value of these genus bacteria was calculated. As shown in Figure 7A, each taxon was powerful enough in discriminating PCD from non-diarrheal patients, in which *Ruminococcus* and *Phascolarctobacterium* were more accurate with an AUC value greater than 0.8. Besides, the combined AUC value of these altered microbes was 0.885 (Figure 7B), which indicated a strong association between these bacteria and PCD.

## Discussion

In this study, alterations in fecal bile acid metabolism were found in PCD patients, accompanied by significant changes in gut microbiota, compared to Non-PCD and HC. These metabolic changes in fecal bile were strongly associated with intestinal bacteria. Based on these findings, several bacterial genera were selected as PCD-linked microbes, such as *Prevotella*, and the ROC results indicated good efficacy in discriminating PCD from Non-PCD by these taxa. Cholecystectomy is a very common surgery performed in hepatobiliary surgery in treating symptomatic gallstone disease (Baron et al., 2015; Lammert et al., 2016). With high incidence, PCD affects a huge amount of outpatients’ quality of life by a large margin for the postoperative alterations in bowel habits (Ruiz-Campos et al., 2019; Ahmad and Faulx, 2020). BAD is accepted as a common cause for PCD according to the results of SeHCAT test (Farrugia et al., 2021). In this article, we deciphered metabolic characteristics of fecal bile acid metabolism among three groups, and found excessive fecal excretion in diarrheal patients. Overflowed primary bile acids directly from biliary ducts could accelerate colonic transit and increase fecal weight in BAD patients (Vijayvargiya et al., 2019a,b)’; our results also demonstrated overabundance of primary bile acids in PCD patients, for example, TCA and TβMCA. Hence, excessive bile acid output in feces could be a reasonable factor for diarrhea after cholecystectomy.

Alterations of bile acid metabolism will influence gut microbiota in IBS-D patients (Jeffery et al., 2020; Zhao et al., 2020) and changes in intestinal flora are involved in gastrointestinal dysfunction or diseases (Vich et al., 2018). As expected, we found declined bacterial community richness and disordered microbial structure in PCD patients. Bile acids are well known to inhibit the growth of many (but not all) bacteria *via* their detergent-like actions disrupting cell wall architecture and stimulating immune system to synthesize antimicrobial peptide cathelicidin through farnesoid X receptor (FXR) or vitamin D receptor (Takeshi et al., 2006; D’Aldebert et al., 2009). A reasonable explanation for decreased microbial richness and disturbed composition in PCD patients might be excessive bile acid content in colon, and another might be the selective pressure and elimination of slower-growing bacteria in direct response to the diarrhea caused by PCD. Small fluctuation of bile acids could trigger a major alteration in bacterial community structure, which profitably helps protect host from pathogens as well, such as *Clostridium difficile* (Buffie et al., 2014). However, in PCD patients, excessive fecal bile content is obviously detrimental for bacterial growth *via* some combination of direct (antimicrobial) and indirect (diarrhea) effects associated with PCD.

Then, we profiled genus bacteria and found several disease-linked microbes, such as cumulative *Prevotella*, and reduced *Alistipes* in PCD patients. Overabundance of *Prevotella* is recognized as adverse (Tong et al., 2020) in IBS-D patients and *Enterococcus* colonization exerts detrimental effects on host health (Arias and Murray, 2012), and both are simultaneously accumulated in diarrheal patients. Besides, *Bacteroides*, *Ruminococcus*, and *Phascolarctobacterium* are reported to be reduced in diarrheal pigs, while they grow back after diarrhea improvement by Gegen Qinlian Decoction (Liu et al., 2019). In our results, a similar variation trend of these microbes was also observed in PCD patients. As described in Zhao’s article that showed decreased *Alistipes* and *Bacteroides* in IBS-D patients, their relevance with PCD may also be significant for their reduced abundance. Moreover, the correlation results and significant AUC values revealed strong associations between these genera and clinical phenotypes of diarrhea. While *Prevotella* and *Sutterella* were identified as detrimental genera by comparing relative abundance of PCD with Non-PCD groups in Li’s work (Li et al., 2021), we analyzed 6 vital bacteria in a larger sample size and pinpointed their strong correlations with diarrheal parameters. However, the causality of these specific bacteria to PCD still requires more experimental verifications.

Furthermore, we demonstrated tight associations between bile acid metabolites and gut microbiota in this study, notably *Prevotella* and TUDCA. Positive correlations indicated that overabundant bile acids favor bacterial growth, for example, *Enterococcus* and CDCA in ulcerative colitis (Yang et al., 2021), but negative associations for detrimental effects, for example, reduced *Alistipes* and *Bacteroides* with total bile in IBS-D patients (Zhao et al., 2020). Besides, *UCG-002*, *Klebsiella*, and *Escherichia-Shigella* were also closely correlated with these bile salts, despite the fact that no significant difference of relative abundance was achieved among three groups. However, effects of bile acids on microbial proliferation or bacterial metabolism on bile acids rely on *ex vivo* single bacteria strain separation and co-culture experiments (Tian et al., 2020; Yan et al., 2021). Inter-crosstalk between intestinal flora and bile acids is bidirectional (Jia et al., 2018). Microbial metabolism of bile acids is essential for their synthesis and transformation, which is dependent on bacterial genes encoding for bile salts metabolism or transformations, as noted in previous studies (Joyce et al., 2014; Wahlstrom et al., 2016; Funabashi et al., 2020; Zhao et al., 2020). Bile salt hydrolase (BSH) in *Lactobacillus* and *Bacteroides* functions to deconjugate taurine and glycine in conjugated bile acids and preserve their adverse effects on gastrointestinal tract (Horackova et al., 2018; Winston and Theriot, 2019). A possible mechanism of elevated fecal tauro-conjugated bile acids may be ascribed to insufficient BSH function in decreased *Lactobacillus* and *Bacteroides*, more than cholecystectomy. Besides, reduced BA-deconjugating enzyme choloylglycine hydrolase by declined *Alistipes* and *Bacteroides*, and decreased 7α-hydroxysteroid dehydrogenase (7α-HSDH) and C-7 epimerization by under-represented *Ruminococcus* might reasonably explain the cumulating conjugated bile salts excretion in PCD patients (Zhao et al., 2020). Besides, *in vitro* microbial experiments of bile microbiota in cholecystitis patients show the metabolizing capability of bile acids of *Enterococcus* (Yan et al., 2021). These might reasonably explain the higher concentration of conjugated bile acids, such as TCA, Tβ-MCA, and TUDCA in feces of PCD patients. Moreover, regulatory effects of microbiota on bile acids synthesis also rely on negative feedback through FXR because bile salts serve as natural ligands for FXR *per se* (Sayin et al., 2013; Wahlstrom et al., 2016).

A co-occurrence network model provides a new perspective to study community structure and function of microorganisms (Qin et al., 2012). It facilitates the identification of dominant bacteria and their associations with others in a particular ecology, which play vital roles in maintaining ecological homeostasis such as microbial diversity (Vazquez-Castellanos et al., 2018). Herein, our results demonstrated a disrupted homeostasis of bacterial ecology. Among these disease-related genera, only *Enterococcus* augmented its associations with other bacteria in PCD, which means that patients’ intestinal environment is suitable for *Enterococcus*. *Bacteroides*, the dominant genus in intestine, is a well-known probiotic, which help maintain gastrointestinal health (Wang et al., 2020). Its reduced abundance and correlation may be a contributing factor but also a potential therapeutical strategy for PCD, and *vice versa* as *Ruminococcus* and *Phascolarctobacterium*. Intriguingly, despite the overabundant *Prevotella* and *Fusicatenibacter* in PCD, their correlations are inversely declined. This result may be attributed to reduced protective effects of decreased probiotics in patients with PCD such as *Lactobacillus*, *Faecalibacterium*, and *Bifidobacterium* (Sanders et al., 2019).

However, there exist two major limitations in this study. Firstly, despite the tight correlation results and significant AUC values between gut microbiota and PCD, we failed to validate the causal relationship of altered microbial composition with diarrhea. Another limitation is that inter-crosstalk between fecal bile salts and bacteria was insufficiently analyzed only through Spearman correlation analysis. Future investigations are supposed to focus on the microbial genome in modifying bile acids by gut microbiota.

Collectively, our results provide evidence that gut microbiota dysbiosis and bile acid metabolism changes are strongly correlated with PCD and ascribe disrupted microbial structure to altered bile acid metabolism in diarrheal patients. Therefore, remodeling patients’ gut microbiota may be a potential therapy for PCD patients.

## Data Availability Statement

The datasets presented in this study can be found in online repositories. The names of the repository/repositories and accession number(s) can be found below: NCBI SRA; PRJNA777105.

## Ethics Statement

The studies involving human participants were reviewed and approved by Ethics Committee of Minhang Hospital, Fudan University. The patients/participants provided their written informed consent to participate in this study.

## Author Contributions

ZZ and XW designed this study. YX conducted almost all experiments and wrote this manuscript. HJ helped collect patient information and samples. JW and QC followed up outpatients and recruited participants. SZ and ZL helped in data processing and R software. All authors contributed to the article and approved the submitted version.

## Conflict of Interest

The authors declare that the research was conducted in the absence of any commercial or financial relationships that could be construed as a potential conflict of interest.

## Publisher’s Note

All claims expressed in this article are solely those of the authors and do not necessarily represent those of their affiliated organizations, or those of the publisher, the editors and the reviewers. Any product that may be evaluated in this article, or claim that may be made by its manufacturer, is not guaranteed or endorsed by the publisher.

## Abbreviations

PCD: post-cholecystectomy diarrhea
BAD: bile acid diarrhea
BSS: Bristol stool score
OTU: operational taxonomic units
TCA: taurocholic acid
dehydroLCA: dehydrolithocholic acid
TLCA: taurolithocholic acid
TUDCA: tauroursodeoxycholic acid
12-DHCA: 12-dehydrocholic acid
CDCA: chenodeoxycholic acid
TβMCA: tauro β-muricholic acid
DCA: deoxycholic acid.
