# Supplementary figures and images for: Disordered Gut Microbiota Correlates With Altered Fecal Bile Acid Metabolism and Post-cholecystectomy Diarrhea
Source: Front Microbiol. 2022 Feb 18;13:800604. doi: 10.3389/fmicb.2022.800604 (PMC8894761; doi:10.3389/fmicb.2022.800604)

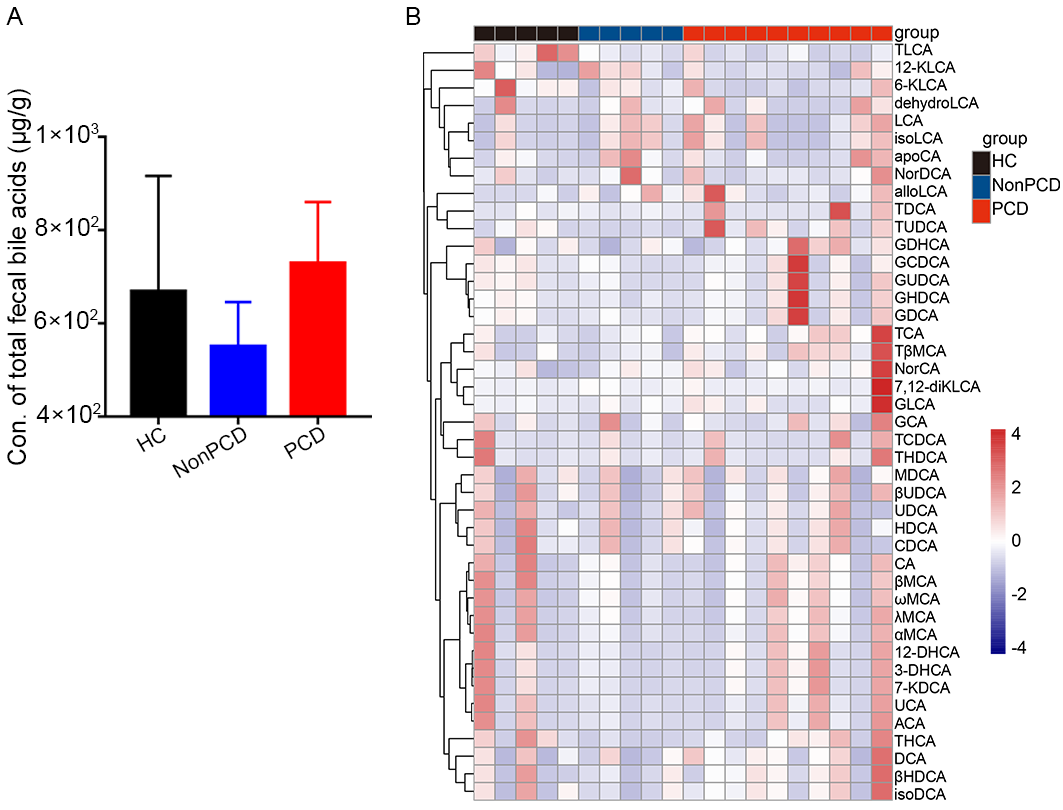

Supplement: Supplementary file 1 [file Image_1.TIF]

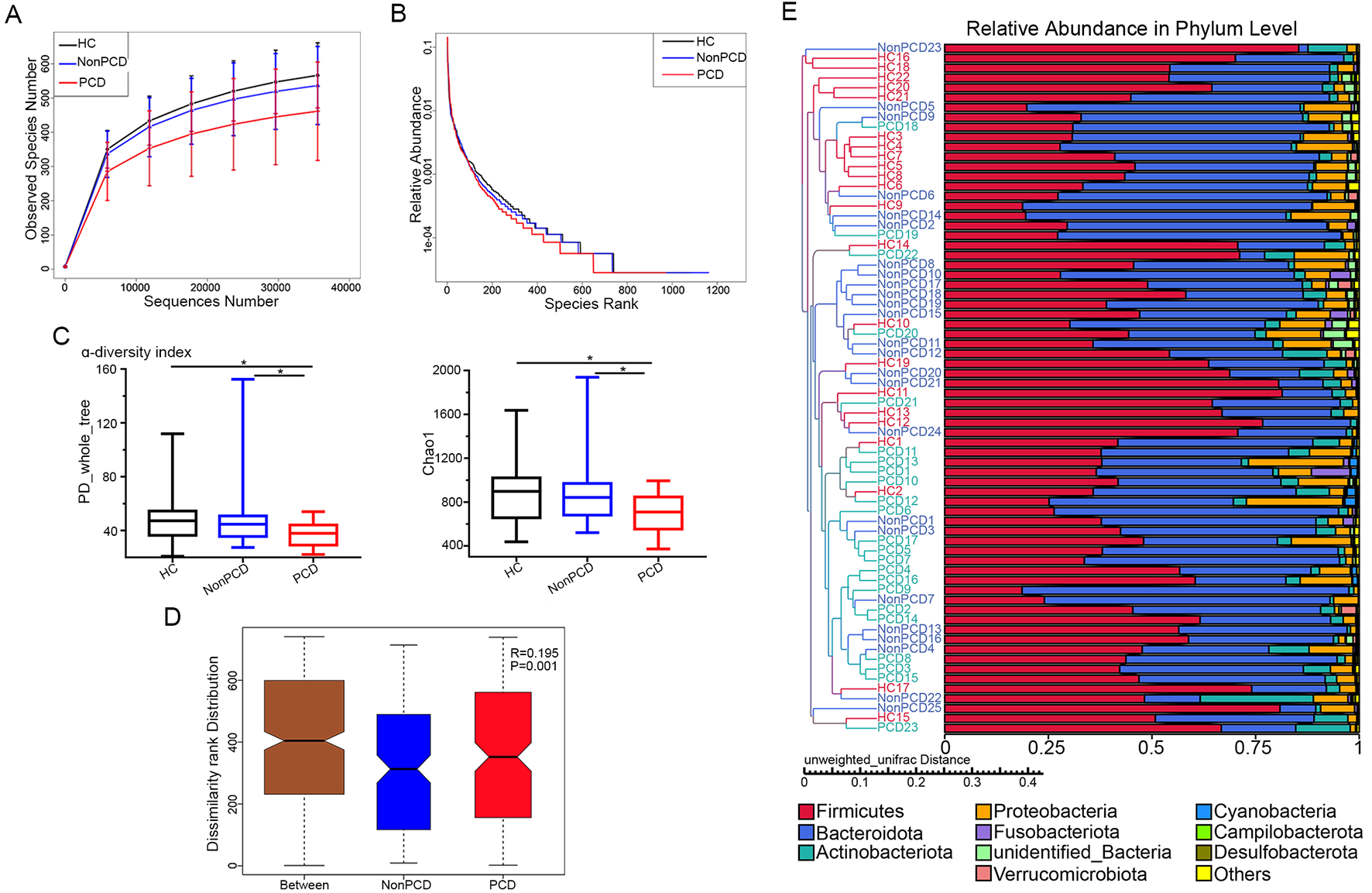

Supplement: Supplementary file 2 [file Image_2.TIF]

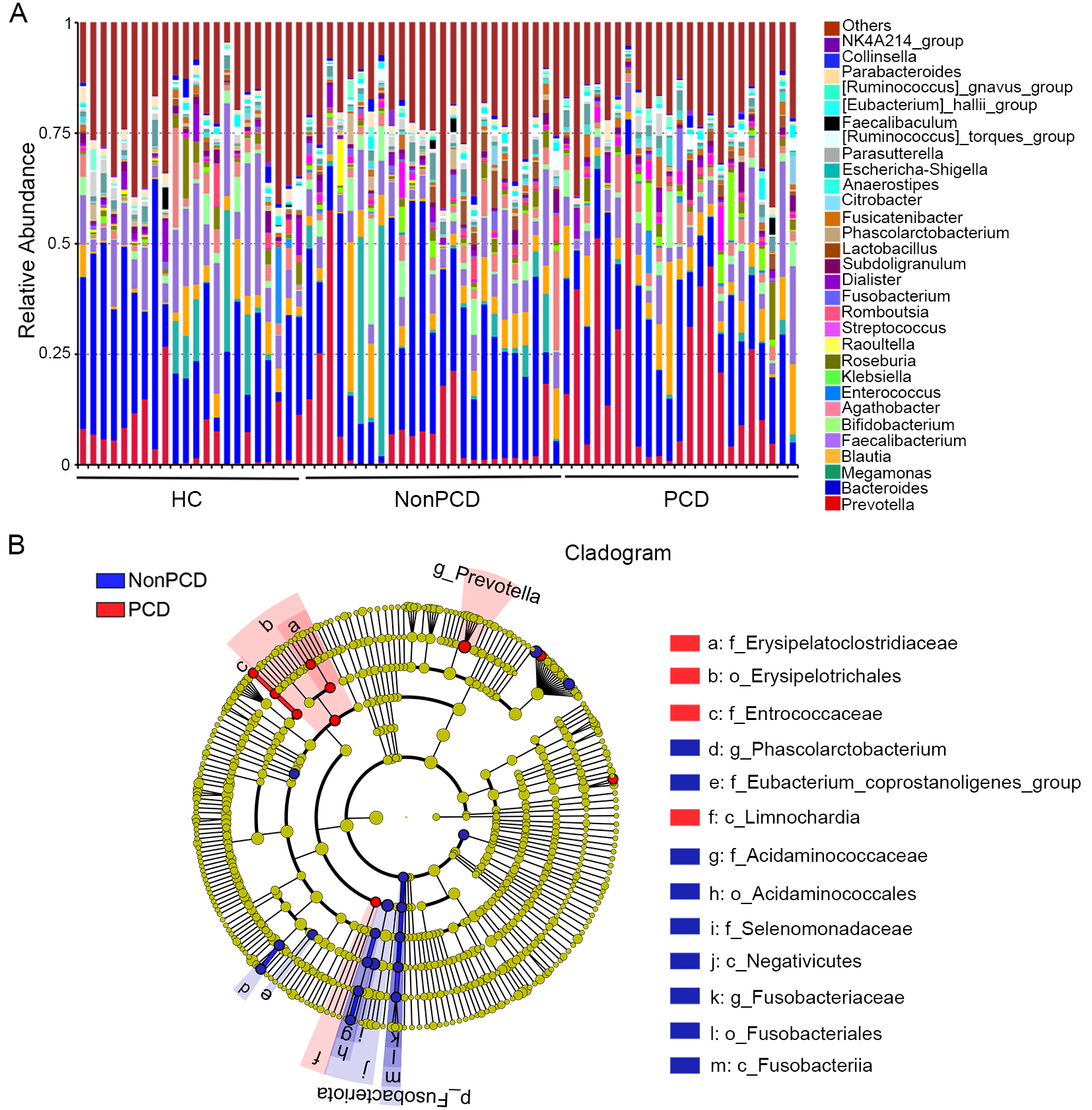

Supplement: Supplementary file 3 [file Image_3.TIF]

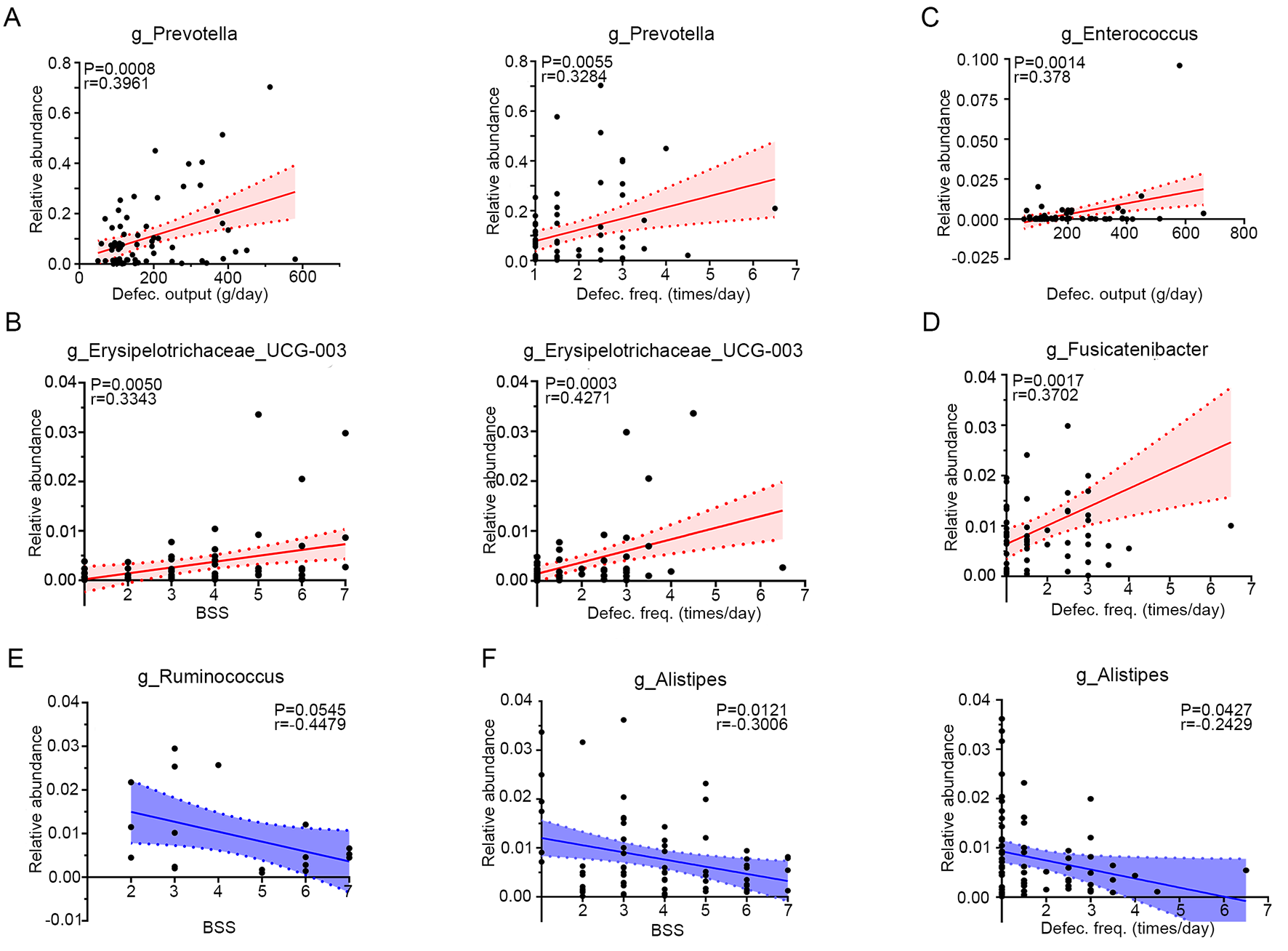

Supplement: Supplementary file 4 [file Image_4.TIF]

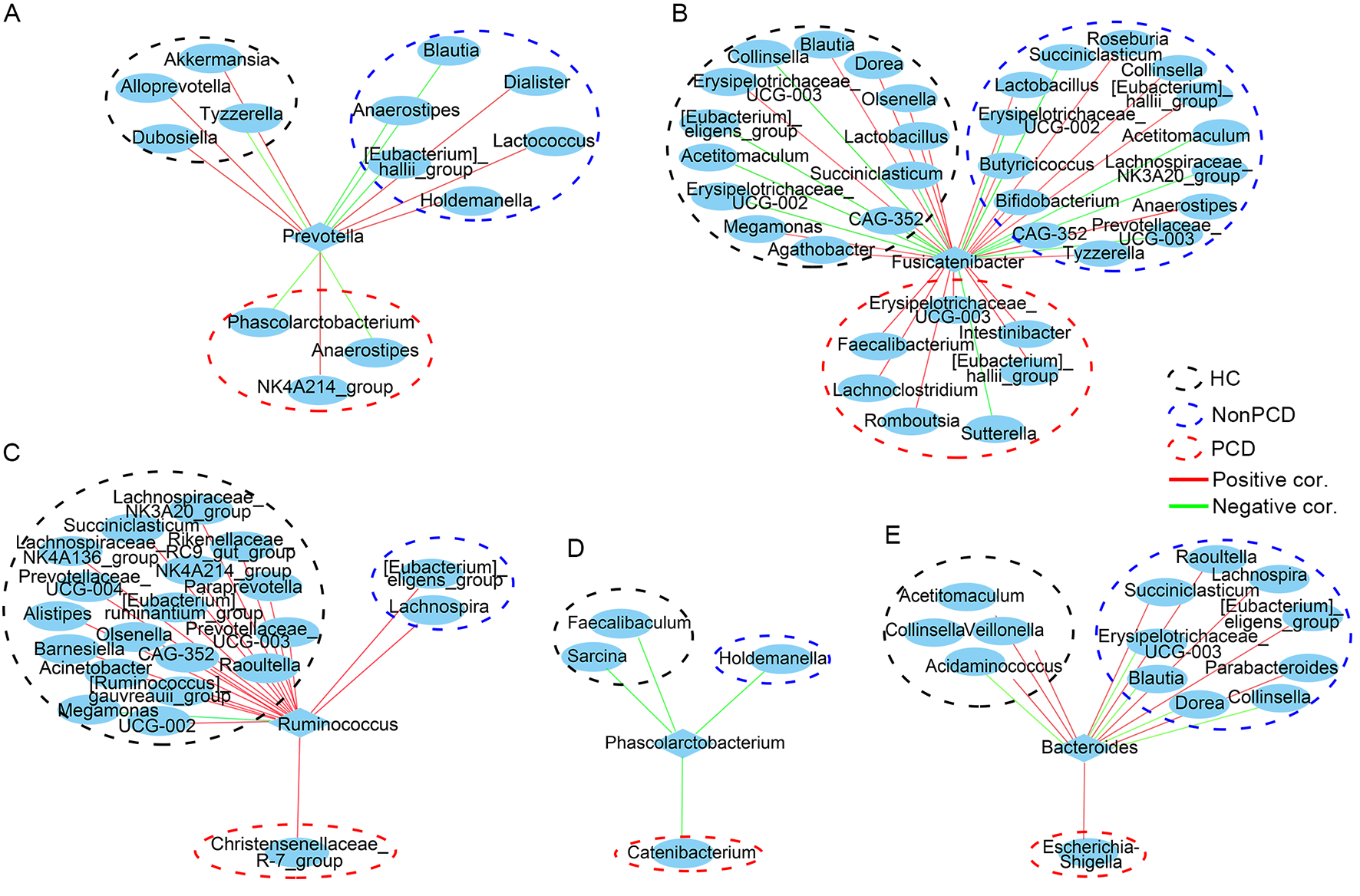

Supplement: Supplementary file 5 [file Image_5.TIF]
