# Supplementary material for: Disordered Gut Microbiota Correlates With Altered Fecal Bile Acid Metabolism and Post-cholecystectomy Diarrhea
Source: Front Microbiol. 2022 Feb 18;13:800604. doi: 10.3389/fmicb.2022.800604 (PMC8894761; doi:10.3389/fmicb.2022.800604)
Supplement: Supplementary file 6 [file Table_1.DOC]

Supplementary figure legends

Figure S1: Alterations of bile acids metabolism in feces from individuals with PCD. (A) Concentration of total bile acids in feces from three grouped individuals, Data are shown as mean ± standard error mean (SEM), n=5 for HC, n=5 NonPCD and n=10 PCD. (B) Heatmap showing distinct fecal concentration of these 43 bile acids detected among three groups.

Figure S2: Changes in gut microbiota composition, richness, and evenness in PCD patients. (A) Rarefaction curves revealing sequencing depth of 16S sequence of gut microbiota in each sample (n=70). (B) Rank abundance curve was applied to intuitively observe richness and evenness of bacteria in each sample (n=70). (C) PD-whole-tree and Chao1 indexes presenting reduced microbial alpha-diversity in PCD group by Box plot. Data are expressed as Mix to Max; n=22 for HC, 25 for NonPCD and 23 for PCD patients. * p<0.05. (D) Boxplot showing dissimilarity rank distribution between NonPCD and PCD groups and the difference was determined by ANOSIM (R=0.195, p=0.001). (E) Unweighted pair-group method with arithmetic means (UPGMA) tree based on unweighted-unifrac distance showing similarity among samples (n=70) in left panel and relative abundance of top 10 bacteria in phylum level of each sample in right panel.

Figure S3: (A) Relative abundance of top 30 bacterial genera in each sample among three groups. (B) Cladogram revealing specific gut bacteria with significant difference and their structures in PCD patients. Yellow nodes denoted taxa with no significance, circle rings from inside to outside denote taxonomic hierarchy from phylum to species, respectively.

Figure S4: Examples of linear correlations between special genera and clinical indexes. Associations between Prevotella and defection output, defection frequency (A). *Erysipelotrichaceae_UCG-003* and BSS, defection frequency (B). *Enterococcus* and defecation output (C). *Fusicatenibacter* and defection frequency (D). *Ruminococcus* and BSS (E). *Alistipes* and BSS, defection frequency (F). Dotted lines showed error bar and area between lines 95% confidence interval, red for positive association and blue for negative. BSS, Bristol stool score, defec. freq., defection frequency, defec. output, defection output.

Figure S5: Co-abundance networks of representative PCD-linked genus microbes with other genera in three groups, respectively. Reduced associations between specific diarrhea-related genera with others in PCD patients, (A) *Prevotella*, (B) *Fusicatenibacter*, (C) *Ruminococcus*, (D) *Phascolarctobacterium*, (E) *Bacteroides*. Correlations between specific genus bacteria and others were calculated and correlation coefficients (P_fdr_<0.05,r>0.6) were regarded as clear links and visualized by Cytoscape v3.8.2. Multiplicity testing were conducted and P value was adjusted using the methods of Benjamini, Hochberg, and Yekutieli to control the false discovery rate (FDR). P_fdr_<0.05 was considered significant. Blue circles represented bacteria in genus, red lines denoted positive correlations and green negative, dotted circles covered genera in each group.

Supplementary Table S1: Basic information of 43 bile acids detected for absolute quantification in the feces of subjects in this study.

| Bile acids (full name) | Abbreviation | Neutral Formula | [MW] | Retention time (min) |
| --- | --- | --- | --- | --- |
| Glycolithocholic acid | GLCA | C_26_H_43_NO_4_ | 433.62 | 8.38 |
| Apocholic acid | apoCA | C_24_H_38_O_4_ | 390.56 | 7.01 |
| 7,12-diketolithocholic acid | 7,12-diKLCA | C_24_H_36_O_5_ | 404.55 | 4.13 |
| Taurolithocholic acid | TLCA | C_26_H_45_NO_5_S | 505.69 | 6.86 |
| Isolithocholic acid | isoLCA | C_24_H_40_O_3_ | 376.57 | 5.16 |
| Murideoxycholic acid | MDCA | C_24_H_40_O_4_ | 392.58 | 7.86 |
| 12-dehydrocholic acid | 12-DHCA | C_24_H_34_O_5_ | 402.52 | 5.30 |
| Tauro β-muricholic acid | TβMCA | C_26_H_45_NO_7_S | 515.7 | 3.33 |
| Glycodehydrocholic acid | GDHCA | C_26_H_37_NO_6_ | 459.58 | 5.38 |
| β-muricholic acid | βMCA | C_24_H_40_O_5_ | 408.57 | 7.86 |
| β-hyodeoxycholic acid | βHDCA | C_24_H_40_O_4_ | 392.57 | 4.19 |
| Norcholic acid | NorCA | C_23_H_38_O_5_ | 394.54 | 4.09 |
| β-ursodeoxycholic acid | βUDCA | C_24_H_40_O_4_ | 392.57 | 11.3 |
| Dehydrolithocholic acid | dehydroLCA | C_24_H_38_O_3_ | 374.56 | 10.9 |
| Allolithocholic acid | alloLCA | C_24_H_40_O_3_ | 376.58 | 11.7 |
| Ursocholic acid | UCA | C_24_H_40_O_5_ | 408.57 | 5.36 |
| Allocholic acid | ACA | C_24_H_40_O_5_ | 408.56 | 5.18 |
| 23-nordeoxycholic acid | NorDCA | C_23_H_38_O_4_ | 378.55 | 5.29 |
| 3-dehydrocholic acid | 3-DHCA | C_24_H_38_O_5_ | 406.56 | 3.63 |
| 6-ketolithocholic acid | 6-KLCA | C_24_H_38_O_4_ | 390.56 | 6.99 |
| 7-ketodeoxycholic acid | 7-KDCA | C_24_H_38_O_4_ | 390.56 | 6.98 |
| Tauroursodeoxycholic acid | TDCA | C_26_H_45_NO_6_S | 499.707 | 4.74 |
| Hyodeoxycholic acid | HDCA | C_24_H_40_O_4_ | 392.578 | 7.86 |
| Deoxycholic acid | DCA | C_24_H_40_O_4_ | 392.58 | 7.86 |
| Taurochenodeoxycholic acid | TCDCA | C_26_H_45_NO_6_S | 499.69 | 4.73 |
| Taurohyodeoxycholic acid | THDCA | C_26_H_44_NO_6_S | 521.69 | 4.73 |
| Taurocholic acid | TCA | C_26_H_45_NO_7_S | 515.7 | 3.49 |
| Tauroursodeoxycholic acid | TUDCA | C_26_H_45_NO_6_S | 499.7 | 4.73 |
| Ursodeoxycholic acid | UDCA | C_24_H_40_O_4_ | 392.578 | 7.86 |
| Chenodeoxycholic acid | CDCA | C_24_H_40_O_4_ | 392.58 | 6.86 |
| Cholic acid | CA | C_24_H_40_O_5_ | 408.57 | 5.39 |
| Glycoursodeoxycholic acid | GUDCA | C_26_H_43_NO_5_ | 449.62 | 4.01 |
| Glycohyodeoxycholic acid | GHDCA | C_26_H_43_NO_5_ | 449.629 | 4.01 |
| Glycochenodeoxycholic acid | GCDCA | C_26_H_43_NO_5_ | 449.62 | 4.01 |
| Glycodeoxycholicacid | GDCA | C_26_H_43_NO_5_ | 449.632 | 4.01 |
| Glycocholic acid | GCA | C_26_H_43_NO_6_ | 465.62 | 3.97 |
| Lithocholic acid | LCA | C_24_H_40_O_3_ | 376.57 | 3.49 |
| Isodeoxycholic acid | isoDCA | C_24_H_40_O_4_ | 392.57 | 5.38 |
| 12-ketolithocholic acid | 12-KLCA | C_24_H_38_O_4_ | 390.5561 | 3.47 |
| ω-muricholic acid | ωMCA | C_24_H_40_O_5_ | 408.57 | 5.34 |
| taurohyocholic acid | THCA | C_26_H_45_NO_7_S | 515.703 | 7.86 |
| λ-Muricholic Acid | λMCA | C_24_H_40_O_5_ | 408.579 | 5.37 |
| α-Muricholic Acid | αMCA | C_26_H_45_NO_7_S | 515.706 | 5.32 |

Supplementary Table S2: Statistic parameters for co-abundance networks of three groups.

| Groups | ND | MD | CC | GD | AD | APL | Pos% | Neg% | ratio |
| --- | --- | --- | --- | --- | --- | --- | --- | --- | --- |
| HC | 7 | 0.377 | 0.517 | 0.090 | 17.608 | 2.719 | 0.754 | 0.246 | 3.065 |
| NonPCD | 6 | 0.239 | 0.477 | 0.068 | 13.388 | 2.505 | 0.634 | 0.366 | 1.732 |
| PCD | 14 | 0.701 | 0.496 | 0.034 | 6.680 | 4.610 | 0.892 | 0.108 | 8.259 |

ND: Network diameter, MD: modularity, CC: Clustering coefficient, GD: graph density, AD: Average degree, APL: average. path. Length between any two nodes, Pos: positive correlations, Neg: negative correlations, Ratio: Pos% / Neg%. HC: healthy control, PCD: post-cholecystectomy, NonPCD: non-PCD.

Supplementary Table S3: Significantly changed microbial taxa in the feces of PCD and NonPCD patients by T-Test.

Supplementary Table S4: Co-occurrence network of genus bacteria in HC, NonPCD and PCD.
