# Supplementary material for: Disordered Gut Microbiota Correlates With Altered Fecal Bile Acid Metabolism and Post-cholecystectomy Diarrhea
Source: Front Microbiol. 2022 Feb 18;13:800604. doi: 10.3389/fmicb.2022.800604 (PMC8894761; doi:10.3389/fmicb.2022.800604)
Supplement: Supplementary file 7 [file Table_2.DOCX]

| Supplementary Table S3: Significantly changed microbial taxa in the feces of PCD and NonPCD patients by t-Test. | | | | | | | | |
| --- | --- | --- | --- | --- | --- | --- | --- | --- |
| Level | Taxa | avg(NonPCD) | sd(NonPCD) | avg(PCD) | sd(PCD) | | P value | q values |
| phylum | p_Fusobacteriota | 0.0090 | 0.0127 | 0.0061 | 0.0192 | | 0.0129 | 0.1446 |
|  | p_unidentified_Bacteria | 0.0100 | 0.0103 | 0.0060 | 0.0109 | | 0.0258 | 0.1573 |
| Class | p_Firmicutes;c_Negativicutes | 0.0810 | 0.1247 | 0.0278 | 0.0218 | | 0.0064 | 0.1725 |
|  | p_Fusobacteriota;c_Fusobacteriia | 0.0090 | 0.0127 | 0.0061 | 0.0192 | | 0.0129 | 0.2561 |
|  | p_unidentified_Bacteria;c_Clostridia | 0.0026 | 0.0024 | 0.0009 | 0.0009 | | 0.0030 | 0.1369 |
| Order | p_Fusobacteriota;c_Fusobacteriia;  o_Fusobacteriales | 0.0090 | 0.0127 | 0.0061 | 0.0192 | | 0.0129 | 0.2476 |
|  | p_Firmicutes;c_Negativicutes;  o_Acidaminococcales | 0.0106 | 0.0158 | 0.0032 | 0.0044 | | 0.0010 | 0.0943 |
|  | p_Proteobacteria;c_Gammaproteobacteria;  o_Burkholderiales | 0.0129 | 0.0095 | 0.0094 | 0.0122 | | 0.0249 | 0.2884 |
|  | p_Firmicutes;c_Bacilli;o_Erysipelotrichales | 0.0103 | 0.0097 | 0.0176 | 0.0133 | | 0.0232 | 0.2804 |
|  | p_Firmicutes;c_Limnochordia;o_MBA03 | 0.0035 | 0.0121 | 0.0055 | 0.0122 | | 0.0141 | 0.2476 |
|  | p_Firmicutes;c_Clostridia;  o_Clostridia_UCG-014 | 0.0070 | 0.0093 | 0.0016 | 0.0027 | | 0.0012 | 0.0943 |
|  | p_Cyanobacteria;c_Cyanobacteriia;  o_Synechococcales | 0.0000 | 0.0000 | 0.0023 | 0.0051 | | 0.0103 | 0.2476 |
|  | p_Firmicutes;c_Clostridia;  o_Caldicoprobacterales | 0.0011 | 0.0038 | 0.0019 | 0.0043 | | 0.0267 | 0.2884 |
| Family | p_Firmicutes;c_Bacilli;o_Lactobacillales;  f_Enterococcaceae | 0.0010 | 0.0021 | 0.0066 | 0.0198 | | 0.0013 | 0.1307 |
|  | p_Fusobacteriota;c_Fusobacteriia;  o_Fusobacteriales;f_Fusobacteriaceae | 0.0089 | 0.0126 | 0.0061 | 0.0192 | | 0.0136 | 0.2595 |
|  | p_Firmicutes;c_Negativicutes;  o_Acidaminococcales;f_Acidaminococcaceae | 0.0106 | 0.0158 | 0.0032 | 0.0044 | | 0.0010 | 0.1307 |
|  | p_Proteobacteria;c_Gammaproteobacteria;  o_Burkholderiales;f_Sutterellaceae | 0.0110 | 0.0066 | 0.0081 | 0.0121 | | 0.0044 | 0.2458 |
|  | p_Firmicutes;c_Clostridia;o_Oscillospirales;  f_[Eubacterium]_coprostanoligenes_group | 0.0101 | 0.0097 | 0.0047 | 0.0033 | | 0.0176 | 0.2595 |
|  | p_Bacteroidota;c_Bacteroidia;o_Bacteroidales;  f_Tannerellaceae | 0.0111 | 0.0080 | 0.0081 | 0.0107 | | 0.0279 | 0.3347 |
|  | p_Firmicutes;c_Bacilli;o_Erysipelotrichales;  f_Erysipelatoclostridiaceae | 0.0041 | 0.0054 | 0.0089 | 0.0096 | | 0.0085 | 0.2595 |
|  | p_Cyanobacteria;c_Cyanobacteriia;  o_Synechococcales;f_Cyanobiaceae | 0.0000 | 0.0000 | 0.0023 | 0.0051 | | 0.0103 | 0.2595 |
|  | p_Firmicutes;c_Clostridia;o_Caldicoprobacterales;  f_Caldicoprobacteraceae | 0.0011 | 0.0038 | 0.0019 | 0.0043 | | 0.0267 | 0.3318 |
| Genus | p_Bacteroidota;c_Bacteroidia;o_Bacteroidales;  f_Prevotellaceae;g_Prevotella | 0.0852 | 0.1263 | 0.1889 | 0.1958 | | 0.0318 | 0.4172 |
|  | p_Firmicutes;c_Bacilli;o_Lactobacillales;  f_Enterococcaceae;g_Enterococcus | 0.0010 | 0.0021 | 0.0066 | 0.0198 | | 0.0013 | 0.1224 |
|  | p_Fusobacteriota;c_Fusobacteriia;  o_Fusobacteriales;f_Fusobacteriaceae;  g_Fusobacterium | 0.0089 | 0.0126 | 0.0060 | 0.0192 | | 0.0118 | 0.3193 |
|  | p_Firmicutes;c_Negativicutes;  o_Acidaminococcales;f_Acidaminococcaceae;  g_Phascolarctobacterium | 0.0100 | 0.0159 | 0.0023 | | 0.0035 | 0.0002 | 0.0602 |
|  | p_Proteobacteria;c_Gammaproteobacteria;  o_Burkholderiales;f_Sutterellaceae;g_Parasutterella | 0.0082 | 0.0067 | 0.0061 | | 0.0123 | 0.0078 | 0.2604 |
|  | p_Bacteroidota;c_Bacteroidia;o_Bacteroidales;  f_Tannerellaceae;g_Parabacteroides | 0.0110 | 0.0079 | 0.0081 | | 0.0107 | 0.0318 | 0.4172 |
|  | p_Firmicutes;c_Bacilli;o_Erysipelotrichales;  f_Erysipelatoclostridiaceae;  g_Erysipelotrichaceae_UCG-003 | 0.0020 | 0.0023 | 0.0062 | | 0.0092 | 0.0258 | 0.4172 |
|  | p_Firmicutes;c_Clostridia;o_Oscillospirales;  f_Ruminococcaceae;g_Ruminococcus | 0.0116 | 0.0088 | 0.0042 | | 0.0037 | 0.0001 | 0.0602 |
|  | p_Firmicutes;c_Bacilli;o_Lactobacillales;  f_Streptococcaceae;g_Lactococcus | 0.0004 | 0.0007 | 0.0021 | | 0.0052 | 0.0497 | 0.5169 |
|  | p_Bacteroidota;c_Bacteroidia;o_Bacteroidales;  f_Prevotellaceae;g_Alloprevotella | 0.0053 | 0.0062 | 0.0024 | | 0.0036 | 0.0300 | 0.4172 |
|  | p_Proteobacteria;c_Gammaproteobacteria;  o_Burkholderiales;f_Sutterellaceae;g_Sutterella | 0.0028 | 0.0052 | 0.0021 | | 0.0014 | 0.0431 | 0.4882 |
|  | p_Bacteroidota;c_Bacteroidia;o_Bacteroidales;  f_Rikenellaceae;g_Rikenellaceae_RC9_gut_group | 0.0013 | 0.0028 | 0.0012 | | 0.0036 | 0.0248 | 0.4172 |
|  | p_Firmicutes;c_Clostridia;o_Caldicoprobacterales;  f_Caldicoprobacteraceae;g_Caldicoprobacter | 0.0011 | 0.0038 | 0.0019 | | 0.0043 | 0.0267 | 0.4172 |
|  | p_Firmicutes;c_Clostridia;o_Lachnospirales;  f_Lachnospiraceae;g_[Eubacterium]_eligens_group | 0.0028 | 0.0028 | 0.0013 | | 0.0011 | 0.0310 | 0.4172 |
|  | p_Firmicutes;c_Bacilli;o_Erysipelotrichales;  f_Erysipelotrichaceae;g_Dubosiella | 0.0003 | 0.0007 | 0.0016 | | 0.0030 | 0.0344 | 0.4172 |
