# Supplementary material for: Disordered Gut Microbiota Correlates With Altered Fecal Bile Acid Metabolism and Post-cholecystectomy Diarrhea
Source: Front Microbiol. 2022 Feb 18;13:800604. doi: 10.3389/fmicb.2022.800604 (PMC8894761; doi:10.3389/fmicb.2022.800604)
Supplement: Supplementary file 8 [file Table_3.DOCX]

| Supplementary Table S4: Co-occurrence network of genus bacteria in HC | | | |
| --- | --- | --- | --- |
| genus1 | genus2 | r | p |
| Ruminofilibacter | Luteimonas | 1 | 0 |
| Ruminofilibacter | Thermobacillus | 1 | 0 |
| Ruminofilibacter | Cycloclasticus | 1 | 0 |
| Ruminofilibacter | Methylotenera | 1 | 0 |
| Luteimonas | Thermobacillus | 1 | 0 |
| Luteimonas | Cycloclasticus | 1 | 0 |
| Luteimonas | Methylotenera | 1 | 0 |
| Thermobacillus | Cycloclasticus | 1 | 0 |
| Thermobacillus | Methylotenera | 1 | 0 |
| Cycloclasticus | Methylotenera | 1 | 0 |
| Cyanobium_PCC-6307 | hgcI_clade | 0.995464853 | 0 |
| CAG-352 | Succiniclasticum | 0.990090834 | 0 |
| Erysipelotrichaceae_UCG-002 | CAG-352 | 0.980181668 | 1.55E-15 |
| Erysipelotrichaceae_UCG-002 | Succiniclasticum | 0.962014864 | 9.65E-13 |
| Blautia | [Eubacterium]_hallii_group | 0.934125898 | 2.11E-10 |
| Faecalibaculum | Dubosiella | 0.933491446 | 2.32E-10 |
| Acetitomaculum | Succiniclasticum | 0.927944383 | 5.05E-10 |
| Paraprevotella | Christensenellaceae_R-7_group | 0.926652684 | 5.99E-10 |
| Faecalibaculum | Helicobacter | 0.921322152 | 1.18E-09 |
| Acetitomaculum | CAG-352 | 0.917642694 | 1.84E-09 |
| Erysipelotrichaceae_UCG-002 | Acetitomaculum | 0.911303194 | 3.76E-09 |
| [Eubacterium]_hallii_group | Butyricicoccus | 0.904722419 | 7.47E-09 |
| Monoglobus | [Eubacterium]_hallii_group | 0.900453065 | 2.25E-06 |
| Helicobacter | Dubosiella | 0.887061411 | 6.95E-06 |
| Saccharopolyspora | Kroppenstedtia | 0.887007916 | 6.95E-06 |
| Butyricicoccus | Blautia | 0.884810841 | 8.03E-06 |
| Ileibacterium | Kroppenstedtia | 0.88419648 | 8.13E-06 |
| Succiniclasticum | Lachnospiraceae_NK3A20_group | 0.883303979 | 8.43E-06 |
| Paraprevotella | UCG-002 | 0.881090734 | 9.73E-06 |
| Ruminococcus | NK4A214_group | 0.880293619 | 1.00E-05 |
| Butyricicoccus | Monoglobus | 0.878847818 | 7.39E-08 |
| Lachnospiraceae_NK3A20_group | CAG-352 | 0.867982141 | 1.66E-07 |
| Succiniclasticum | Olsenella | 0.862287553 | 3.42E-05 |
| Lachnospiraceae_NK3A20_group | Erysipelotrichaceae_UCG-002 | 0.860321222 | 3.80E-05 |
| Christensenellaceae_R-7_group | UCG-002 | 0.859644203 | 3.86E-05 |
| Lachnospiraceae_NK3A20_group | Acetitomaculum | 0.852801713 | 5.86E-05 |
| Prevotellaceae_UCG-003 | Rikenellaceae_RC9_gut_group | 0.85187738 | 6.05E-05 |
| CAG-352 | Olsenella | 0.847684885 | 7.64E-05 |
| Acidaminococcus | Raoultella | 0.845872901 | 8.31E-05 |
| Intestinibacter | Romboutsia | 0.843087396 | 9.58E-05 |
| Dorea | [Eubacterium]_hallii_group | 0.842195109 | 9.85E-05 |
| Olsenella | Erysipelotrichaceae_UCG-002 | 0.83600275 | 0.000134 |
| Acetitomaculum | Parasutterella | 0.832442436 | 0.00016 |
| Blautia | Monoglobus | 0.831403591 | 1.64E-06 |
| Catenibacterium | Holdemanella | 0.81596992 | 0.000356 |
| Acidaminococcus | Klebsiella | 0.80942037 | 0.000481 |
| Lachnospiraceae_NK3A20_group | Olsenella | 0.804717939 | 0.000588 |
| Megasphaera | Acidaminococcus | 0.804120584 | 6.50E-06 |
| Acidaminococcus | Megasphaera | 0.804120584 | 0.000593 |
| Raoultella | Erysipelotrichaceae_UCG-002 | 0.802339001 | 7.06E-06 |
| Rikenellaceae_RC9_gut_group | Paraprevotella | 0.801795629 | 0.000635 |
| Blautia | Dorea | 0.800903731 | 7.54E-06 |
| Collinsella | Fusicatenibacter | 0.800001568 | 0.000662 |
| Collinsella | Dorea | 0.799661594 | 7.98E-06 |
| CAG-352 | Raoultella | 0.795440128 | 0.000786 |
| Raoultella | Succiniclasticum | 0.794060353 | 1.03E-05 |
| Dorea | Butyricicoccus | 0.79299636 | 0.000839 |
| Alistipes | Paraprevotella | 0.792755159 | 1.09E-05 |
| [Eubacterium]_hallii_group | Collinsella | 0.784136103 | 1.57E-05 |
| Escherichia-Shigella | Klebsiella | 0.779785432 | 0.00134 |
| Agathobacter | Monoglobus | 0.778876055 | 1.95E-05 |
| Butyricicoccus | Collinsella | 0.776804918 | 0.001459 |
| Lachnospiraceae_NK3A20_group | Prevotellaceae_UCG-004 | 0.77457882 | 2.32E-05 |
| Christensenellaceae_R-7_group | Alistipes | 0.77266312 | 0.001622 |
| Ileibacterium | Saccharopolyspora | 0.77244389 | 2.52E-05 |
| Olsenella | Acetitomaculum | 0.770733258 | 0.00171 |
| Escherichia-Shigella | [Ruminococcus]_gnavus_group | 0.761017071 | 3.91E-05 |
| Butyricicoccus | Agathobacter | 0.759457933 | 0.002521 |
| Christensenellaceae_R-7_group | Rikenellaceae_RC9_gut_group | 0.758383033 | 4.31E-05 |
| Collinsella | Blautia | 0.758134443 | 0.002578 |
| Agathobacter | Fusicatenibacter | 0.756070017 | 4.70E-05 |
| Barnesiella | Alistipes | 0.751632081 | 0.003181 |
| Parasutterella | Succiniclasticum | 0.749286526 | 5.99E-05 |
| Monoglobus | Collinsella | 0.748727734 | 0.003346 |
| UCG-005 | Prevotellaceae_UCG-003 | 0.748470572 | 6.16E-05 |
| Acidaminococcus | Erysipelotrichaceae_UCG-002 | 0.747617317 | 0.003408 |
| Parasutterella | CAG-352 | 0.746554396 | 6.59E-05 |
| Succiniclasticum | Veillonella | 0.741299573 | 0.004142 |
| Erysipelotrichaceae_UCG-002 | Veillonella | 0.738566672 | 8.67E-05 |
| CAG-352 | Veillonella | 0.738566672 | 0.004441 |
| Christensenellaceae_R-7_group | NK4A214_group | 0.738209575 | 0.004445 |
| Dorea | Monoglobus | 0.735875706 | 0.004753 |
| Sarcina | Faecalibaculum | 0.735533955 | 0.004755 |
| Helicobacter | Ileibacterium | 0.734441447 | 0.004877 |
| Acidaminococcus | CAG-352 | 0.733603871 | 0.00496 |
| Olsenella | Raoultella | 0.73321108 | 0.004972 |
| Rikenellaceae_RC9_gut_group | Alloprevotella | 0.731635313 | 0.005168 |
| Prevotellaceae_UCG-003 | Paraprevotella | 0.731396138 | 0.005168 |
| Lactobacillus | Fusicatenibacter | 0.730921892 | 0.000112 |
| Streptococcus | Blautia | 0.730302204 | 0.005229 |
| Blautia | Agathobacter | 0.730095991 | 0.000115 |
| Ruminococcus | [Ruminococcus]_gauvreauii_group | 0.728042955 | 0.000123 |
| Prevotellaceae_UCG-004 | CAG-352 | 0.727973374 | 0.005435 |
| UCG-002 | NK4A214_group | 0.72783738 | 0.005435 |
| Anaerostipes | Bifidobacterium | 0.727683732 | 0.005435 |
| Acidaminococcus | Succiniclasticum | 0.725195804 | 0.005828 |
| Paraprevotella | NK4A214_group | 0.724804717 | 0.005846 |
| Prevotellaceae_UCG-003 | Alistipes | 0.723398793 | 0.006011 |
| Erysipelotrichaceae_UCG-002 | Parasutterella | 0.723331295 | 0.006011 |
| Ruminococcus | Christensenellaceae_R-7_group | 0.721265208 | 0.000152 |
| Rikenellaceae_RC9_gut_group | Prevotellaceae_UCG-004 | 0.720031027 | 0.000158 |
| Ruminococcus | Lachnospiraceae_NK4A136_group | 0.719209154 | 0.000162 |
| Ruminococcus | [Eubacterium]_ruminantium_group | 0.718764038 | 0.000164 |
| Coprococcus | Dorea | 0.71674671 | 0.000175 |
| Lactococcus | Dubosiella | 0.716338168 | 0.000177 |
| Streptococcus | Butyricicoccus | 0.713922649 | 0.00019 |
| [Ruminococcus]_gnavus_group | Veillonella | 0.713114754 | 0.000195 |
| Raoultella | Acetitomaculum | 0.712691236 | 0.000198 |
| Christensenellaceae_R-7_group | Prevotellaceae_UCG-003 | 0.711740768 | 0.000203 |
| Klebsiella | Raoultella | 0.711389471 | 0.000206 |
| Raoultella | Rikenellaceae_RC9_gut_group | 0.710662824 | 0.00021 |
| Raoultella | Acinetobacter | 0.710200856 | 0.000213 |
| Rikenellaceae_RC9_gut_group | Succiniclasticum | 0.709894095 | 0.000215 |
| Agathobacter | [Eubacterium]_hallii_group | 0.709641648 | 0.000217 |
| Holdemanella | Intestinibacter | 0.708214793 | 0.000226 |
| Agathobacter | Collinsella | 0.707780737 | 0.000229 |
| NK4A214_group | Family_XIII_AD3011_group | 0.706757135 | 0.000236 |
| [Ruminococcus]_gauvreauii_group | Olsenella | 0.705391953 | 0.000246 |
| Faecalibaculum | Ileibacterium | 0.705221508 | 0.000247 |
| Ruminococcus | Lachnospiraceae_NK3A20_group | 0.705080713 | 0.000248 |
| Romboutsia | Turicibacter | 0.704803436 | 0.00025 |
| Turicibacter | Romboutsia | 0.704803436 | 0.008237 |
| Megasphaera | Shuttleworthia | 0.704624863 | 0.000251 |
| Ruminococcus | UCG-002 | 0.704121965 | 0.000255 |
| Christensenellaceae_R-7_group | [Eubacterium]_ruminantium_group | 0.701916449 | 0.000272 |
| CAG-352 | Rikenellaceae_RC9_gut_group | 0.701615447 | 0.008676 |
| Sarcina | Catenibacterium | 0.701102469 | 0.008685 |
| [Eubacterium]_ruminantium_group | Olsenella | 0.700500894 | 0.008776 |
| Acetitomaculum | Veillonella | 0.699908215 | 0.008866 |
| Prevotellaceae_UCG-004 | Succiniclasticum | 0.698664326 | 0.008886 |
| [Eubacterium]_ventriosum_group | Lachnospiraceae_NK3A20_group | 0.696408415 | 0.009352 |
| Sarcina | Dubosiella | 0.695140986 | 0.009569 |
| Escherichia-Shigella | CAG-352 | 0.694447791 | 0.000336 |
| Ruminococcus | Succiniclasticum | 0.694447791 | 0.000336 |
| Prevotellaceae_UCG-004 | Erysipelotrichaceae_UCG-002 | 0.693911508 | 0.00969 |
| Acinetobacter | Paraprevotella | 0.693795582 | 0.00969 |
| [Eubacterium]_hallii_group | Lachnoclostridium | 0.693243554 | 0.000347 |
| Erysipelotrichaceae_UCG-002 | Escherichia-Shigella | 0.693082112 | 0.009764 |
| UCG-005 | Paraprevotella | 0.691806763 | 0.010055 |
| UCG-005 | UCG-002 | 0.691547199 | 0.010066 |
| Prevotellaceae_UCG-004 | NK4A214_group | 0.69105917 | 0.010103 |
| Fusicatenibacter | Megamonas | 0.690981247 | 0.010103 |
| Dorea | Holdemanella | 0.690071669 | 0.010184 |
| Christensenellaceae_R-7_group | UCG-005 | 0.69004569 | 0.00038 |
| [Ruminococcus]_gnavus_group | Acetitomaculum | 0.68843431 | 0.000397 |
| Blautia | Lachnoclostridium | 0.686053077 | 0.000423 |
| Paraprevotella | Prevotellaceae_UCG-001 | 0.685930641 | 0.000425 |
| Raoultella | [Ruminococcus]_gauvreauii_group | 0.684484912 | 0.000442 |
| Faecalibaculum | Turicibacter | 0.682709922 | 0.000463 |
| Succiniclasticum | Klebsiella | 0.68215668 | 0.012118 |
| Escherichia-Shigella | Veillonella | 0.681356041 | 0.000481 |
| Ruminococcus | Rikenellaceae_RC9_gut_group | 0.681153993 | 0.000483 |
| Tyzzerella | Anaerostipes | 0.680974813 | 0.01229 |
| Klebsiella | CAG-352 | 0.680791001 | 0.000488 |
| NK4A214_group | Rikenellaceae_RC9_gut_group | 0.680013031 | 0.000498 |
| NK4A214_group | Lachnospiraceae_NK4A136_group | 0.679661125 | 0.000503 |
| Sarcina | Cyanobium_PCC-6307 | 0.679544042 | 0.012401 |
| Succiniclasticum | Escherichia-Shigella | 0.679425322 | 0.012401 |
| UBA1819 | Alistipes | 0.677987023 | 0.012744 |
| Sarcina | hgcI_clade | 0.676462209 | 0.013125 |
| Succiniclasticum | [Ruminococcus]_gauvreauii_group | 0.675519028 | 0.013313 |
| Klebsiella | Olsenella | 0.675004727 | 0.000568 |
| Tyzzerella | Megamonas | 0.67454601 | 0.013444 |
| CAG-352 | [Ruminococcus]_gnavus_group | 0.674343483 | 0.013446 |
| Ruminococcus | Paraprevotella | 0.672709378 | 0.000603 |
| Ruminococcus | Olsenella | 0.672589683 | 0.000605 |
| UCG-002 | Alistipes | 0.671372106 | 0.014222 |
| Erysipelotrichaceae_UCG-002 | Klebsiella | 0.671231247 | 0.014222 |
| Butyricicoccus | Coprococcus | 0.670242801 | 0.014359 |
| Olsenella | Rikenellaceae_RC9_gut_group | 0.669161859 | 0.014618 |
| Kroppenstedtia | Holdemanella | 0.667557865 | 0.015059 |
| Succiniclasticum | [Ruminococcus]_gnavus_group | 0.667511229 | 0.015059 |
| [Eubacterium]_hallii_group | Streptococcus | 0.667421242 | 0.015059 |
| Sarcina | Helicobacter | 0.667164687 | 0.015085 |
| Kroppenstedtia | Helicobacter | 0.666496812 | 0.000707 |
| Lachnospiraceae_NK3A20_group | Parasutterella | 0.665992033 | 0.015351 |
| Alistipes | NK4A214_group | 0.665725579 | 0.015351 |
| Acidaminococcus | Shuttleworthia | 0.665227168 | 0.01541 |
| Sarcina | Holdemanella | 0.665128632 | 0.01541 |
| Haemophilus | Klebsiella | 0.664971858 | 0.01541 |
| Collinsella | Roseburia | 0.664782067 | 0.01541 |
| Butyricicoccus | Lachnoclostridium | 0.664596273 | 0.01541 |
| Prevotellaceae_UCG-003 | Prevotellaceae_UCG-004 | 0.664471581 | 0.000743 |
| Paraprevotella | Saccharopolyspora | 0.663069964 | 0.00077 |
| Escherichia-Shigella | Haemophilus | 0.662146998 | 0.000788 |
| Romboutsia | Dorea | 0.661486322 | 0.000801 |
| Lachnospiraceae_NK4A136_group | [Ruminococcus]_gauvreauii_group | 0.661486322 | 0.000801 |
| UCG-002 | Acinetobacter | 0.660254127 | 0.000826 |
| Erysipelotrichaceae_UCG-002 | Rikenellaceae_RC9_gut_group | 0.660222205 | 0.000826 |
| Akkermansia | Family_XIII_AD3011_group | 0.657948417 | 0.000874 |
| [Ruminococcus]_gauvreauii_group | CAG-352 | 0.657760186 | 0.000878 |
| Rikenellaceae_RC9_gut_group | Acetitomaculum | 0.657410494 | 0.000885 |
| Agathobacter | Dorea | 0.656876615 | 0.000897 |
| Barnesiella | Megasphaera | 0.656738249 | 0.0009 |
| Veillonella | Lachnospiraceae_NK3A20_group | 0.656672441 | 0.000901 |
| [Eubacterium]_ruminantium_group | NK4A214_group | 0.656262818 | 0.016928 |
| Collinsella | Megamonas | 0.656109737 | 0.016928 |
| Lachnospiraceae_NK3A20_group | Raoultella | 0.656035101 | 0.016928 |
| Rikenellaceae_RC9_gut_group | Acinetobacter | 0.655842721 | 0.016928 |
| Rikenellaceae_RC9_gut_group | Alistipes | 0.65548236 | 0.016928 |
| Sarcina | Enterococcus | 0.65546868 | 0.016928 |
| Dorea | Fusicatenibacter | 0.655182178 | 0.016928 |
| Ruminofilibacter | Caldicoprobacter | 0.654818592 | 0.000943 |
| Luteimonas | Caldicoprobacter | 0.654818592 | 0.000943 |
| Thermobacillus | Caldicoprobacter | 0.654818592 | 0.000943 |
| Caldicoprobacter | Cycloclasticus | 0.654818592 | 0.000943 |
| Caldicoprobacter | Methylotenera | 0.654818592 | 0.000943 |
| Erysipelotrichaceae_UCG-002 | [Ruminococcus]_gauvreauii_group | 0.652295927 | 0.001002 |
| Christensenellaceae_R-7_group | Acinetobacter | 0.649911002 | 0.001061 |
| Fusobacterium | Escherichia-Shigella | 0.649322202 | 0.001076 |
| Olsenella | Prevotellaceae_UCG-004 | 0.648920912 | 0.001086 |
| Acinetobacter | NK4A214_group | 0.648557863 | 0.019136 |
| [Eubacterium]_hallii_group | Romboutsia | 0.6472421 | 0.019665 |
| Haemophilus | Succiniclasticum | 0.647014466 | 0.001136 |
| Ruminococcus | Prevotellaceae_UCG-003 | 0.646315315 | 0.001155 |
| Ruminococcus | Barnesiella | 0.646278979 | 0.001156 |
| Holdemanella | Romboutsia | 0.645896928 | 0.019916 |
| Christensenellaceae_R-7_group | Barnesiella | 0.644761794 | 0.0203 |
| Erysipelotrichaceae_UCG-002 | [Ruminococcus]_gnavus_group | 0.644281565 | 0.020326 |
| Megasphaera | Alistipes | 0.644231697 | 0.020326 |
| Kroppenstedtia | Faecalibaculum | 0.644053054 | 0.020336 |
| [Ruminococcus]_torques_group | Megamonas | 0.643708685 | 0.020351 |
| Blautia | Romboutsia | 0.642937956 | 0.001249 |
| Prevotellaceae_UCG-004 | Acetitomaculum | 0.642721445 | 0.020605 |
| Ruminococcus | CAG-352 | 0.642551988 | 0.001261 |
| Sarcina | Kroppenstedtia | 0.640235901 | 0.021464 |
| Holdemanella | [Eubacterium]_hallii_group | 0.640101386 | 0.021464 |
| Parasutterella | [Eubacterium]_eligens_group | 0.640011328 | 0.001337 |
| NK4A214_group | Klebsiella | 0.639751553 | 0.021517 |
| Olsenella | Escherichia-Shigella | 0.639382832 | 0.021535 |
| Family_XIII_AD3011_group | UCG-002 | 0.639257858 | 0.021535 |
| Family_XIII_AD3011_group | Sarcina | 0.638800523 | 0.001374 |
| [Eubacterium]_ventriosum_group | Veillonella | 0.638315934 | 0.021777 |
| Haemophilus | Olsenella | 0.637931861 | 0.001402 |
| Rikenellaceae_RC9_gut_group | UCG-002 | 0.637226976 | 0.021948 |
| Ruminococcus | Raoultella | 0.636656496 | 0.021956 |
| Haemophilus | Lachnospira | 0.63662054 | 0.001444 |
| Lachnospiraceae_NK3A20_group | [Ruminococcus]_gauvreauii_group | 0.636209326 | 0.022005 |
| Haemophilus | CAG-352 | 0.63608286 | 0.001462 |
| Lachnospira | Erysipelotrichaceae_UCG-002 | 0.634537085 | 0.022698 |
| Rikenellaceae_RC9_gut_group | Lachnospiraceae_NK3A20_group | 0.634273937 | 0.001523 |
| Megasphaera | Raoultella | 0.633737566 | 0.022825 |
| [Ruminococcus]_gnavus_group | Olsenella | 0.63219289 | 0.001596 |
| Citrobacter | Klebsiella | 0.631638519 | 0.023617 |
| Bacteroides | Veillonella | 0.631073547 | 0.001636 |
| Anaerostipes | Faecalibacterium | 0.629378632 | 0.024519 |
| UCG-002 | Prevotellaceae_UCG-003 | 0.62911977 | 0.001709 |
| Alistipes | Raoultella | 0.628669765 | 0.024673 |
| Olsenella | Acidaminococcus | 0.627272614 | 0.00178 |
| UCG-005 | Rikenellaceae_RC9_gut_group | 0.626988304 | 0.025268 |
| [Ruminococcus]_gnavus_group | Lachnospiraceae_NK3A20_group | 0.626881317 | 0.001795 |
| [Eubacterium]_ruminantium_group | Lachnospiraceae_NK4A136_group | 0.626502449 | 0.025401 |
| Barnesiella | [Eubacterium]_ruminantium_group | 0.625822397 | 0.001838 |
| Acetitomaculum | Acidaminococcus | 0.625663047 | 0.001844 |
| Barnesiella | Paraprevotella | 0.624822519 | 0.001878 |
| Raoultella | Paraprevotella | 0.624445187 | 0.001894 |
| Roseburia | Butyricicoccus | 0.623941276 | 0.001915 |
| Klebsiella | Megasphaera | 0.623869676 | 0.001917 |
| Lachnospira | Succiniclasticum | 0.623608567 | 0.001928 |
| [Eubacterium]_ruminantium_group | [Ruminococcus]_gauvreauii_group | 0.622915326 | 0.026437 |
| [Eubacterium]_eligens_group | Acetitomaculum | 0.622869137 | 0.026437 |
| Dubosiella | Ileibacterium | 0.622716665 | 0.026446 |
| Lachnospira | CAG-352 | 0.622242502 | 0.001986 |
| UCG-005 | Intestinibacter | 0.622241186 | 0.026563 |
| Blautia | Fusicatenibacter | 0.621682665 | 0.002011 |
| Butyricicoccus | Anaerostipes | 0.620904054 | 0.027025 |
| Ruminococcus | Alistipes | 0.62055336 | 0.02715 |
| Acinetobacter | Lachnospiraceae_NK4A136_group | 0.61849691 | 0.002153 |
| Helicobacter | Turicibacter | 0.618433268 | 0.027954 |
| Collinsella | Coprococcus | 0.618388765 | 0.002158 |
| Lactobacillus | Megamonas | 0.618183429 | 0.027997 |
| Lachnospira | Lachnospiraceae_NK3A20_group | 0.616565412 | 0.002243 |
| Monoglobus | Romboutsia | 0.615710677 | 0.0292 |
| Haemophilus | Erysipelotrichaceae_UCG-002 | 0.615586097 | 0.00229 |
| Olsenella | Acinetobacter | 0.615312663 | 0.0292 |
| Family_XIII_AD3011_group | Prevotellaceae_UCG-001 | 0.615276629 | 0.002305 |
| Ruminococcus | Acinetobacter | 0.614638696 | 0.002337 |
| [Ruminococcus]_gauvreauii_group | Acetitomaculum | 0.614172096 | 0.00236 |
| Prevotellaceae_UCG-001 | UCG-002 | 0.613352432 | 0.030156 |
| Romboutsia | Coprococcus | 0.612429476 | 0.002448 |
| Coprococcus | Erysipelotrichaceae_UCG-003 | 0.611472387 | 0.030935 |
| Raoultella | Prevotellaceae_UCG-003 | 0.61135082 | 0.002503 |
| Acinetobacter | Erysipelotrichaceae_UCG-002 | 0.61033066 | 0.031429 |
| Barnesiella | Prevotellaceae_UCG-003 | 0.60937251 | 0.002608 |
| Megasphaera | Escherichia-Shigella | 0.608598161 | 0.032226 |
| Paraprevotella | Family_XIII_AD3011_group | 0.608078246 | 0.002679 |
| [Eubacterium]_eligens_group | Barnesiella | 0.607822889 | 0.032257 |
| Alistipes | Acidaminococcus | 0.607789526 | 0.002695 |
| Prevotella | Akkermansia | 0.607417765 | 0.002716 |
| Paraprevotella | Prevotellaceae_UCG-004 | 0.606965978 | 0.002741 |
| [Eubacterium]_ruminantium_group | UCG-002 | 0.606830034 | 0.032558 |
| Ruminococcus | Prevotellaceae_UCG-004 | 0.60590496 | 0.002802 |
| Fusicatenibacter | Erysipelotrichaceae_UCG-003 | 0.605821071 | 0.002806 |
| Prevotella | Alloprevotella | 0.605497132 | 0.002825 |
| Faecalibaculum | Lactococcus | 0.604899338 | 0.00286 |
| Dorea | Intestinibacter | 0.6048616 | 0.033463 |
| Enterococcus | Kroppenstedtia | 0.603588193 | 0.002937 |
| Lachnospiraceae_NK4A136_group | Alloprevotella | 0.603572379 | 0.034046 |
| Escherichia-Shigella | Acetitomaculum | 0.603514172 | 0.002942 |
| Butyricicoccus | Romboutsia | 0.602259983 | 0.034747 |
| Prevotella | Dubosiella | 0.600893047 | 0.003102 |
| [Ruminococcus]_torques_group | Dorea | 0.600282486 | 0.00314 |
| Acidaminococcus | Escherichia-Shigella | 0.60025734 | 0.035785 |
| Prevotellaceae_UCG-004 | [Eubacterium]_ventriosum_group | 0.600179139 | 0.003147 |
| CAG-352 | Romboutsia | -0.601921589 | 0.034897 |
| [Eubacterium]_hallii_group | Lachnospira | -0.602375951 | 0.00301 |
| CAG-352 | Butyricicoccus | -0.604312974 | 0.033754 |
| Acetitomaculum | Dorea | -0.607289698 | 0.002723 |
| Succiniclasticum | Romboutsia | -0.608070618 | 0.03224 |
| Veillonella | Dubosiella | -0.608116973 | 0.002677 |
| Rikenellaceae_RC9_gut_group | Anaerostipes | -0.608476134 | 0.032226 |
| Dubosiella | Tyzzerella | -0.608928108 | 0.002633 |
| Phascolarctobacterium | Faecalibaculum | -0.609947919 | 0.002577 |
| Agathobacter | Succiniclasticum | -0.61114137 | 0.002514 |
| Helicobacter | Tyzzerella | -0.611365969 | 0.030935 |
| Veillonella | Dorea | -0.612037323 | 0.002468 |
| Olsenella | Agathobacter | -0.61281735 | 0.030413 |
| Megamonas | Parasutterella | -0.615343824 | 0.002302 |
| [Ruminococcus]_gnavus_group | Romboutsia | -0.615884681 | 0.0292 |
| Veillonella | Ileibacterium | -0.619591229 | 0.002103 |
| Lachnospiraceae_NK3A20_group | Romboutsia | -0.619908926 | 0.02737 |
| Escherichia-Shigella | Monoglobus | -0.62016382 | 0.002077 |
| Acetitomaculum | Romboutsia | -0.620902182 | 0.027025 |
| Erysipelotrichaceae_UCG-002 | Butyricicoccus | -0.622066802 | 0.001994 |
| Olsenella | [Eubacterium]_hallii_group | -0.623962715 | 0.026101 |
| Raoultella | [Eubacterium]_hallii_group | -0.62441751 | 0.001895 |
| Dorea | Erysipelotrichaceae_UCG-002 | -0.625657664 | 0.025561 |
| Collinsella | Lachnospiraceae_NK3A20_group | -0.625864871 | 0.001836 |
| Acinetobacter | Megamonas | -0.628143631 | 0.024805 |
| Fusobacterium | Dorea | -0.628289172 | 0.001741 |
| Acinetobacter | Bifidobacterium | -0.628674213 | 0.024673 |
| Bifidobacterium | Raoultella | -0.630381208 | 0.001662 |
| [Ruminococcus]_gnavus_group | Faecalibaculum | -0.631300282 | 0.02372 |
| Acetitomaculum | Monoglobus | -0.632197425 | 0.001596 |
| Erysipelotrichaceae_UCG-003 | Dialister | -0.632947388 | 0.023158 |
| Escherichia-Shigella | Holdemanella | -0.634129195 | 0.001528 |
| Ruminococcus | Megamonas | -0.634267543 | 0.022698 |
| Agathobacter | Fusobacterium | -0.636313133 | 0.001455 |
| Tyzzerella | Lachnospiraceae_NK4A136_group | -0.636656051 | 0.021956 |
| Faecalibacterium | Rikenellaceae_RC9_gut_group | -0.636656496 | 0.001443 |
| [Eubacterium]_eligens_group | Fusicatenibacter | -0.637288237 | 0.021948 |
| Raoultella | Collinsella | -0.637632647 | 0.001412 |
| Helicobacter | [Ruminococcus]_gnavus_group | -0.638120135 | 0.0218 |
| Agathobacter | CAG-352 | -0.63845495 | 0.001385 |
| NK4A214_group | Megamonas | -0.639475945 | 0.021535 |
| Roseburia | Olsenella | -0.641797875 | 0.001283 |
| Butyricicoccus | Escherichia-Shigella | -0.642010164 | 0.020791 |
| Agathobacter | Erysipelotrichaceae_UCG-002 | -0.642551988 | 0.001261 |
| Parabacteroides | [Ruminococcus]_torques_group | -0.643885934 | 0.020341 |
| Megamonas | Raoultella | -0.644322162 | 0.00121 |
| Monoglobus | Raoultella | -0.644825303 | 0.0203 |
| CAG-352 | Dorea | -0.646148636 | 0.001159 |
| Erysipelotrichaceae_UCG-002 | Romboutsia | -0.649064143 | 0.019045 |
| Holdemanella | Haemophilus | -0.652869319 | 0.000988 |
| Tyzzerella | Alloprevotella | -0.655370994 | 0.016928 |
| Megamonas | Acetitomaculum | -0.657828642 | 0.000876 |
| Acidaminococcus | Collinsella | -0.658921344 | 0.016626 |
| [Ruminococcus]_gnavus_group | Collinsella | -0.658930748 | 0.000853 |
| Olsenella | Collinsella | -0.660008838 | 0.016327 |
| Dorea | Succiniclasticum | -0.660492316 | 0.000821 |
| Lactobacillus | Acetitomaculum | -0.661270853 | 0.000805 |
| [Eubacterium]_hallii_group | Acetitomaculum | -0.663426675 | 0.000763 |
| Erysipelotrichaceae_UCG-002 | Megamonas | -0.663448903 | 0.015605 |
| Klebsiella | Collinsella | -0.663650523 | 0.000759 |
| Lachnospiraceae_NK4A136_group | Megamonas | -0.665894057 | 0.015351 |
| Blautia | Succiniclasticum | -0.669865568 | 0.000649 |
| Ileibacterium | [Ruminococcus]_gnavus_group | -0.670804054 | 0.014224 |
| [Ruminococcus]_gnavus_group | Dorea | -0.67081099 | 0.000633 |
| Collinsella | Parasutterella | -0.671195082 | 0.014222 |
| [Eubacterium]_hallii_group | Lachnospiraceae_NK3A20_group | -0.674992939 | 0.000568 |
| Veillonella | [Eubacterium]_hallii_group | -0.676096425 | 0.013182 |
| Megamonas | CAG-352 | -0.677445716 | 0.000533 |
| Veillonella | Faecalibaculum | -0.678442835 | 0.012659 |
| Holdemanella | Veillonella | -0.680125028 | 0.000497 |
| CAG-352 | Blautia | -0.687619396 | 0.010759 |
| Fusicatenibacter | Acetitomaculum | -0.690666599 | 0.000373 |
| Veillonella | Romboutsia | -0.69615602 | 0.009359 |
| Megamonas | Succiniclasticum | -0.698440934 | 0.0003 |
| Fusicatenibacter | Erysipelotrichaceae_UCG-002 | -0.698544828 | 0.000299 |
| Erysipelotrichaceae_UCG-002 | Blautia | -0.698544828 | 0.008886 |
| Succiniclasticum | Monoglobus | -0.698742129 | 0.008886 |
| Veillonella | Helicobacter | -0.699304806 | 0.000293 |
| Succiniclasticum | Fusicatenibacter | -0.701276186 | 0.008685 |
| Erysipelotrichaceae_UCG-002 | Monoglobus | -0.702840324 | 0.000264 |
| CAG-352 | Fusicatenibacter | -0.704007544 | 0.008267 |
| Escherichia-Shigella | [Eubacterium]_hallii_group | -0.70681439 | 0.000235 |
| Olsenella | Fusicatenibacter | -0.707004056 | 0.008026 |
| Megamonas | Rikenellaceae_RC9_gut_group | -0.7083451 | 0.000225 |
| Tyzzerella | Prevotella | -0.708699672 | 0.007925 |
| Lactobacillus | Parasutterella | -0.711337547 | 0.000206 |
| Dorea | Escherichia-Shigella | -0.713357837 | 0.007477 |
| Phascolarctobacterium | Sarcina | -0.7160797 | 0.000178 |
| Monoglobus | CAG-352 | -0.716500971 | 0.007009 |
| Escherichia-Shigella | Collinsella | -0.748516319 | 6.15E-05 |
| Alloprevotella | Megamonas | -0.749447341 | 0.003346 |
| [Eubacterium]_hallii_group | Succiniclasticum | -0.769281168 | 2.86E-05 |
| Erysipelotrichaceae_UCG-002 | [Eubacterium]_hallii_group | -0.774067807 | 0.001565 |
| Collinsella | CAG-352 | -0.776559075 | 2.14E-05 |
| Succiniclasticum | Collinsella | -0.779980041 | 0.00134 |
| Collinsella | Erysipelotrichaceae_UCG-002 | -0.783401006 | 1.62E-05 |
| CAG-352 | [Eubacterium]_hallii_group | -0.783641084 | 0.001191 |
| Collinsella | Acetitomaculum | -0.82729115 | 2.05E-06 |
| Lactobacillus | [Eubacterium]_eligens_group | -0.841063483 | 9.47E-07 |

| Supplementary Table S4: Co-occurrence network of genus bacteria in NonPCD patients | | | |
| --- | --- | --- | --- |
| genus1 | genus2 | r | p |
| Amphibacillus | Fastidiosipila | 1 | 0 |
| Proteiniphilum | Amphibacillus | 0.996528 | 0 |
| Proteiniphilum | Fastidiosipila | 0.996528 | 0 |
| Caldicoprobacter | Ruminofilibacter | 0.984906 | 0 |
| Erysipelotrichaceae_UCG-002 | Succiniclasticum | 0.982677 | 0 |
| Prevotellaceae_UCG-003 | Lachnospiraceae_NK3A20_group | 0.942641 | 1.51E-09 |
| Erysipelotrichaceae_UCG-002 | Acetitomaculum | 0.929727 | 1.85E-11 |
| Succiniclasticum | Acetitomaculum | 0.912102 | 1.21E-07 |
| Erysipelotrichaceae_UCG-002 | CAG-352 | 0.911866 | 2.29E-10 |
| Prevotellaceae_UCG-003 | Acetitomaculum | 0.903634 | 2.91E-07 |
| Cycloclasticus | Methylotenera | 0.882217 | 5.52E-09 |
| Lachnospiraceae_NK3A20_group | Acetitomaculum | 0.878869 | 2.97E-06 |
| CAG-352 | Succiniclasticum | 0.867048 | 2.06E-08 |
| Anaerostipes | Blautia | 0.866705 | 7.19E-06 |
| Blautia | Butyricicoccus | 0.862308 | 3.01E-08 |
| Prevotellaceae_UCG-003 | Succiniclasticum | 0.843768 | 3.47E-05 |
| Erysipelotrichaceae_UCG-002 | Lachnospiraceae_NK3A20_group | 0.838652 | 1.65E-07 |
| Prevotellaceae_UCG-004 | Prevotellaceae_UCG-003 | 0.838163 | 4.49E-05 |
| Lachnospiraceae_NK3A20_group | Succiniclasticum | 0.834665 | 2.14E-07 |
| Thermobacillus | Luteimonas | 0.833554 | 5.31E-05 |
| Erysipelotrichaceae_UCG-002 | Prevotellaceae_UCG-003 | 0.832647 | 2.43E-07 |
| Butyricicoccus | Anaerostipes | 0.832468 | 5.31E-05 |
| Acetitomaculum | CAG-352 | 0.827957 | 3.26E-07 |
| Monoglobus | Agathobacter | 0.82602 | 7.27E-05 |
| Catenibacterium | CAG-352 | 0.825255 | 3.84E-07 |
| Prevotellaceae_UCG-004 | Lachnospiraceae_NK3A20_group | 0.816889 | 0.000115 |
| Alistipes | Barnesiella | 0.814088 | 7.39E-07 |
| Christensenellaceae_R-7_group | Rikenellaceae_RC9_gut_group | 0.810352 | 9.11E-07 |
| CAG-352 | Porphyromonas | 0.795809 | 0.000313 |
| Agathobacter | Roseburia | 0.792308 | 2.36E-06 |
| Lachnoclostridium | [Eubacterium]_hallii_group | 0.785151 | 0.000497 |
| Lactobacillus | Roseburia | 0.783763 | 0.000516 |
| Blautia | [Eubacterium]_hallii_group | 0.782843 | 3.74E-06 |
| Coprococcus | Agathobacter | 0.776901 | 0.000671 |
| Turicibacter | Helicobacter | 0.773282 | 5.83E-06 |
| Bacteroides | [Eubacterium]_eligens_group | 0.771302 | 6.38E-06 |
| Acetitomaculum | Prevotellaceae_UCG-004 | 0.763014 | 9.19E-06 |
| Lachnoclostridium | Blautia | 0.756154 | 0.001389 |
| Alloprevotella | Turicibacter | 0.755727 | 1.25E-05 |
| Tyzzerella | Anaerostipes | 0.755633 | 0.001389 |
| Intestinibacter | Turicibacter | 0.753707 | 1.36E-05 |
| Christensenellaceae_R-7_group | UCG-002 | 0.752072 | 0.001465 |
| Alloprevotella | Helicobacter | 0.751946 | 1.46E-05 |
| [Eubacterium]_hallii_group | Romboutsia | 0.751683 | 0.001465 |
| Acinetobacter | Shuttleworthia | 0.749351 | 1.63E-05 |
| Collinsella | Bifidobacterium | 0.749038 | 0.001566 |
| [Eubacterium]_hallii_group | Dorea | 0.746728 | 1.81E-05 |
| Lactococcus | Holdemanella | 0.745502 | 0.001702 |
| Barnesiella | Helicobacter | 0.744975 | 1.94E-05 |
| Catenibacterium | Erysipelotrichaceae_UCG-002 | 0.742509 | 0.001825 |
| Romboutsia | Clostridium_sensu_stricto_1 | 0.742351 | 2.15E-05 |
| Shuttleworthia | [Ruminococcus]_gauvreauii_group | 0.739161 | 0.001914 |
| Raoultella | Acetitomaculum | 0.738134 | 2.53E-05 |
| CAG-352 | Lachnospiraceae_NK3A20_group | 0.73758 | 0.001914 |
| Olsenella | Succiniclasticum | 0.737185 | 2.63E-05 |
| Caldicoprobacter | Amphibacillus | 0.737154 | 2.63E-05 |
| Caldicoprobacter | Fastidiosipila | 0.737154 | 2.63E-05 |
| Prevotellaceae_UCG-004 | CAG-352 | 0.736319 | 0.001914 |
| Lachnoclostridium | Butyricicoccus | 0.736154 | 2.73E-05 |
| Olsenella | Erysipelotrichaceae_UCG-002 | 0.736013 | 0.001914 |
| Acetitomaculum | Olsenella | 0.735642 | 2.79E-05 |
| Monoglobus | Butyricicoccus | 0.734796 | 0.001914 |
| Ruminofilibacter | Thermobacillus | 0.734595 | 2.90E-05 |
| Proteiniphilum | Caldicoprobacter | 0.734595 | 0.001914 |
| Alistipes | Helicobacter | 0.729926 | 3.45E-05 |
| Erysipelotrichaceae_UCG-002 | Porphyromonas | 0.728992 | 3.58E-05 |
| Acidaminococcus | Erysipelotrichaceae_UCG-002 | 0.726293 | 0.002502 |
| Rikenellaceae_RC9_gut_group | Lachnospiraceae_NK3A20_group | 0.724636 | 4.19E-05 |
| Ileibacterium | Saccharopolyspora | 0.721688 | 4.66E-05 |
| Proteiniphilum | Sarcina | 0.721688 | 4.66E-05 |
| Monoglobus | Lachnospiraceae_UCG-004 | 0.721109 | 4.76E-05 |
| Tyzzerella | Megamonas | 0.721065 | 0.002751 |
| Agathobacter | Butyricicoccus | 0.720769 | 4.82E-05 |
| Prevotella | Dialister | 0.720523 | 4.86E-05 |
| Anaerostipes | Lachnoclostridium | 0.718215 | 5.28E-05 |
| Erysipelotrichaceae_UCG-003 | Dorea | 0.713378 | 6.25E-05 |
| Succiniclasticum | Acidaminococcus | 0.712546 | 6.43E-05 |
| Acetitomaculum | Acidaminococcus | 0.709441 | 7.16E-05 |
| Enterococcus | Sutterella | 0.70693 | 7.79E-05 |
| Clostridium_sensu_stricto_1 | Turicibacter | 0.705701 | 8.12E-05 |
| Monoglobus | Roseburia | 0.705543 | 0.004 |
| Porphyromonas | Succiniclasticum | 0.704627 | 8.42E-05 |
| Olsenella | NK4A214_group | 0.703882 | 0.00413 |
| Agathobacter | Dorea | 0.703483 | 8.74E-05 |
| Lachnospiraceae_UCG-004 | Agathobacter | 0.703233 | 0.00413 |
| Catenibacterium | Succiniclasticum | 0.703077 | 8.86E-05 |
| Lachnospiraceae_NK3A20_group | Christensenellaceae_R-7_group | 0.702631 | 0.004151 |
| Alistipes | Lachnospiraceae_NK4A136_group | 0.6995 | 9.97E-05 |
| Enterococcus | Holdemanella | 0.699013 | 0.000101 |
| Intestinibacter | Helicobacter | 0.698795 | 0.000102 |
| Coprococcus | Subdoligranulum | 0.698749 | 0.004294 |
| Anaerostipes | [Eubacterium]_hallii_group | 0.698538 | 0.000103 |
| Lactobacillus | Coprococcus | 0.698248 | 0.000104 |
| Raoultella | Succiniclasticum | 0.697924 | 0.000105 |
| Prevotellaceae_UCG-003 | CAG-352 | 0.696865 | 0.004414 |
| Lachnospiraceae_NK4A136_group | [Eubacterium]_eligens_group | 0.696556 | 0.00011 |
| Lactobacillus | Agathobacter | 0.696037 | 0.004458 |
| Blautia | Monoglobus | 0.69438 | 0.000118 |
| Lactobacillus | Helicobacter | 0.691452 | 0.000129 |
| Acinetobacter | Succiniclasticum | 0.690738 | 0.000132 |
| Alistipes | Akkermansia | 0.689789 | 0.000136 |
| Erysipelotrichaceae_UCG-002 | Acinetobacter | 0.688313 | 0.000143 |
| Dorea | Romboutsia | 0.688089 | 0.004874 |
| Raoultella | Erysipelotrichaceae_UCG-002 | 0.685679 | 0.000155 |
| Monoglobus | Anaerostipes | 0.685467 | 0.005033 |
| Blautia | Agathobacter | 0.684615 | 0.00016 |
| Collinsella | Fusicatenibacter | 0.682195 | 0.005358 |
| Romboutsia | Turicibacter | 0.6795 | 0.000187 |
| Prevotellaceae_UCG-003 | Rikenellaceae_RC9_gut_group | 0.678489 | 0.005663 |
| Bifidobacterium | Fusicatenibacter | 0.672822 | 0.000229 |
| Enterococcus | Streptococcus | 0.671771 | 0.000236 |
| Dialister | Lactococcus | 0.671202 | 0.00024 |
| Prevotellaceae_UCG-003 | Raoultella | 0.670089 | 0.006926 |
| Olsenella | Lachnospiraceae_NK3A20_group | 0.668156 | 0.000262 |
| Collinsella | [Eubacterium]_hallii_group | 0.66795 | 0.00724 |
| Coprococcus | Helicobacter | 0.66777 | 0.000265 |
| [Ruminococcus]_torques_group | Romboutsia | 0.666667 | 0.007432 |
| Acetitomaculum | Catenibacterium | 0.664929 | 0.000288 |
| Bacteroides | Parabacteroides | 0.663077 | 0.000303 |
| Erysipelotrichaceae_UCG-002 | Shuttleworthia | 0.662807 | 0.000306 |
| Lactobacillus | Intestinibacter | 0.661663 | 0.000316 |
| Sarcina | Amphibacillus | 0.661547 | 0.000317 |
| Sarcina | Fastidiosipila | 0.661547 | 0.000317 |
| Porphyromonas | Acetitomaculum | 0.660816 | 0.000323 |
| Turicibacter | Barnesiella | 0.660566 | 0.008292 |
| [Ruminococcus]_torques_group | Dorea | 0.660508 | 0.000326 |
| Catenibacterium | Porphyromonas | 0.658885 | 0.008588 |
| Coprococcus | Monoglobus | 0.658255 | 0.000348 |
| Intestinibacter | Barnesiella | 0.657875 | 0.008742 |
| NK4A214_group | Erysipelotrichaceae_UCG-002 | 0.657158 | 0.000358 |
| NK4A214_group | Dialister | 0.656786 | 0.008799 |
| Turicibacter | Lachnospiraceae_UCG-004 | 0.656714 | 0.000363 |
| Olsenella | Sutterella | 0.656533 | 0.008799 |
| Faecalibaculum | Dubosiella | 0.654885 | 0.000382 |
| Acidaminococcus | Catenibacterium | 0.651712 | 0.009706 |
| Erysipelotrichaceae_UCG-002 | Prevotellaceae_UCG-004 | 0.651396 | 0.00042 |
| Lactobacillus | Fusicatenibacter | 0.649413 | 0.000444 |
| CAG-352 | Acidaminococcus | 0.648279 | 0.000457 |
| [Eubacterium]_hallii_group | Butyricicoccus | 0.648202 | 0.000458 |
| Bacteroides | Lachnospira | 0.647557 | 0.000466 |
| Bifidobacterium | Intestinibacter | 0.646403 | 0.000481 |
| Prevotella | Lactococcus | 0.645606 | 0.000491 |
| Olsenella | Raoultella | 0.64491 | 0.011017 |
| Agathobacter | [Eubacterium]_hallii_group | 0.642816 | 0.000529 |
| Succiniclasticum | NK4A214_group | 0.640633 | 0.012115 |
| Bacteroides | Raoultella | 0.640259 | 0.000566 |
| Fusicatenibacter | Tyzzerella | 0.640093 | 0.000569 |
| Prevotellaceae_UCG-003 | Olsenella | 0.639844 | 0.012148 |
| NK4A214_group | Lachnospiraceae_NK3A20_group | 0.639232 | 0.000582 |
| Dorea | Blautia | 0.638445 | 0.01238 |
| Dialister | UCG-002 | 0.6382 | 0.000598 |
| Tyzzerella | Roseburia | 0.638044 | 0.012401 |
| Agathobacter | [Ruminococcus]_torques_group | 0.634737 | 0.000654 |
| Christensenellaceae_R-7_group | NK4A214_group | 0.634278 | 0.013438 |
| Olsenella | CAG-352 | 0.633727 | 0.000671 |
| Coprococcus | [Eubacterium]_hallii_group | 0.631883 | 0.014106 |
| Fusicatenibacter | Anaerostipes | 0.631397 | 0.000712 |
| Dorea | Anaerostipes | 0.630485 | 0.014377 |
| NK4A214_group | Acetitomaculum | 0.628834 | 0.00076 |
| Fusicatenibacter | Roseburia | 0.628198 | 0.014984 |
| Agathobacter | Anaerostipes | 0.627813 | 0.00078 |
| UCG-002 | NK4A214_group | 0.62707 | 0.015291 |
| Agathobacter | Turicibacter | 0.622522 | 0.00089 |
| Lachnoclostridium | Bifidobacterium | 0.621538 | 0.016767 |
| Agathobacter | Helicobacter | 0.621456 | 0.000914 |
| Prevotellaceae_UCG-003 | Acinetobacter | 0.620949 | 0.016848 |
| Veillonella | Olsenella | 0.620013 | 0.000947 |
| Sutterella | Haemophilus | 0.619873 | 0.017037 |
| Roseburia | Coprococcus | 0.619442 | 0.00096 |
| Shuttleworthia | Acetitomaculum | 0.618827 | 0.017348 |
| Ruminococcus | Lachnospira | 0.618315 | 0.000987 |
| Prevotella | Holdemanella | 0.617572 | 0.001005 |
| Holdemanella | Amphibacillus | 0.617335 | 0.001011 |
| Holdemanella | Fastidiosipila | 0.617335 | 0.001011 |
| Erysipelotrichaceae_UCG-003 | Collinsella | 0.616481 | 0.017768 |
| Coprococcus | Turicibacter | 0.616185 | 0.001039 |
| [Eubacterium]_hallii_group | Fusicatenibacter | 0.614467 | 0.018386 |
| Succiniclasticum | Shuttleworthia | 0.613323 | 0.001113 |
| Prevotellaceae_UCG-004 | Succiniclasticum | 0.613209 | 0.01875 |
| NK4A214_group | Catenibacterium | 0.611851 | 0.001153 |
| Tyzzerella | Bifidobacterium | 0.611475 | 0.01933 |
| Acinetobacter | [Ruminococcus]_gauvreauii_group | 0.611346 | 0.001167 |
| UCG-005 | Christensenellaceae_R-7_group | 0.611186 | 0.019336 |
| Roseburia | Helicobacter | 0.610854 | 0.001181 |
| Proteiniphilum | Holdemanella | 0.61032 | 0.019495 |
| Lachnospira | Prevotellaceae_UCG-001 | 0.60975 | 0.001212 |
| Alloprevotella | Barnesiella | 0.609315 | 0.019534 |
| Roseburia | Butyricicoccus | 0.609231 | 0.001227 |
| Amphibacillus | Ruminofilibacter | 0.609176 | 0.019534 |
| Fastidiosipila | Ruminofilibacter | 0.609176 | 0.019534 |
| Monoglobus | Dorea | 0.608511 | 0.001248 |
| Lachnospira | Lachnospiraceae_NK4A136_group | 0.607198 | 0.020263 |
| Ruminofilibacter | Proteiniphilum | 0.606616 | 0.001305 |
| Thermobacillus | Caldicoprobacter | 0.606616 | 0.001305 |
| Christensenellaceae_R-7_group | Acetitomaculum | 0.60658 | 0.001306 |
| Roseburia | Anaerostipes | 0.605886 | 0.001328 |
| Fusicatenibacter | Butyricicoccus | 0.605886 | 0.001328 |
| UCG-002 | Prevotellaceae_UCG-001 | 0.605133 | 0.001351 |
| Anaerostipes | Bifidobacterium | 0.605116 | 0.020527 |
| Bifidobacterium | Streptococcus | 0.604732 | 0.001364 |
| Acetitomaculum | Acinetobacter | 0.604069 | 0.020835 |
| Romboutsia | Collinsella | 0.602772 | 0.001427 |
| Acetitomaculum | Rikenellaceae_RC9_gut_group | 0.602137 | 0.021495 |
| Bacteroides | Succiniclasticum | 0.602045 | 0.001452 |
| NK4A214_group | CAG-352 | 0.60174 | 0.001462 |
| Dorea | Coprococcus | 0.601695 | 0.021498 |
| Olsenella | Helicobacter | -0.60009 | 0.001518 |
| Butyricicoccus | Olsenella | -0.60164 | 0.001465 |
| UCG-002 | Lachnoclostridium | -0.60319 | 0.001414 |
| Lachnoclostridium | UCG-002 | -0.60319 | 0.021129 |
| Bacteroides | Blautia | -0.60385 | 0.001392 |
| Coprococcus | Olsenella | -0.60426 | 0.001379 |
| Shuttleworthia | Intestinibacter | -0.60522 | 0.020527 |
| Roseburia | Erysipelotrichaceae_UCG-002 | -0.60535 | 0.001345 |
| Lactobacillus | Acinetobacter | -0.60633 | 0.001314 |
| Haemophilus | Helicobacter | -0.60681 | 0.001299 |
| NK4A214_group | [Ruminococcus]_torques_group | -0.60897 | 0.019563 |
| Roseburia | Lachnospiraceae_NK3A20_group | -0.60936 | 0.001224 |
| Butyricicoccus | UCG-002 | -0.61012 | 0.019495 |
| Blautia | Veillonella | -0.61022 | 0.001199 |
| Raoultella | Faecalibacterium | -0.61062 | 0.019463 |
| Fusicatenibacter | CAG-352 | -0.61272 | 0.001129 |
| [Eubacterium]_eligens_group | Collinsella | -0.61328 | 0.01875 |
| Succiniclasticum | Tyzzerella | -0.61541 | 0.001059 |
| Prevotellaceae_UCG-003 | [Eubacterium]_hallii_group | -0.61606 | 0.017821 |
| [Ruminococcus]_torques_group | [Eubacterium]_ventriosum_group | -0.61716 | 0.001015 |
| Erysipelotrichaceae_UCG-003 | Acetitomaculum | -0.61769 | 0.001002 |
| Turicibacter | [Eubacterium]_ventriosum_group | -0.61841 | 0.000985 |
| Shuttleworthia | Collinsella | -0.61861 | 0.017371 |
| Lactobacillus | Prevotellaceae_UCG-004 | -0.62026 | 0.000941 |
| Bacteroides | Dorea | -0.62036 | 0.000939 |
| Lachnoclostridium | Acetitomaculum | -0.62104 | 0.000923 |
| Prevotella | Anaerostipes | -0.62178 | 0.000906 |
| Megasphaera | Acinetobacter | -0.62223 | 0.000896 |
| Erysipelotrichaceae_UCG-002 | Anaerostipes | -0.62225 | 0.016643 |
| [Eubacterium]_hallii_group | CAG-352 | -0.62236 | 0.000893 |
| Monoglobus | Erysipelotrichaceae_UCG-002 | -0.62371 | 0.016298 |
| Erysipelotrichaceae_UCG-002 | Tyzzerella | -0.62423 | 0.000853 |
| Succiniclasticum | Roseburia | -0.62516 | 0.015848 |
| Bacteroides | Collinsella | -0.62664 | 0.000803 |
| Fusicatenibacter | Lachnospiraceae_NK3A20_group | -0.62666 | 0.000803 |
| Prevotellaceae_UCG-003 | Romboutsia | -0.62857 | 0.014905 |
| Sutterella | Lachnospiraceae_UCG-004 | -0.62878 | 0.000761 |
| Prevotellaceae_UCG-003 | Roseburia | -0.63148 | 0.014106 |
| Megamonas | Catenibacterium | -0.63149 | 0.00071 |
| Dorea | Erysipelotrichaceae_UCG-002 | -0.63268 | 0.013883 |
| Coprococcus | Erysipelotrichaceae_UCG-002 | -0.63678 | 0.00062 |
| Succiniclasticum | Monoglobus | -0.63748 | 0.01253 |
| Megamonas | Shuttleworthia | -0.63885 | 0.000588 |
| Turicibacter | Sutterella | -0.63892 | 0.012304 |
| Anaerostipes | Succiniclasticum | -0.63986 | 0.000572 |
| Erysipelotrichaceae_UCG-003 | Parabacteroides | -0.64294 | 0.011489 |
| Coprococcus | Succiniclasticum | -0.64339 | 0.000521 |
| Erysipelotrichaceae_UCG-003 | Erysipelotrichaceae_UCG-002 | -0.64494 | 0.0005 |
| Anaerostipes | UCG-002 | -0.64717 | 0.000471 |
| Phascolarctobacterium | Holdemanella | -0.64772 | 0.000464 |
| Dorea | Succiniclasticum | -0.6492 | 0.000446 |
| Acetitomaculum | Megamonas | -0.6531 | 0.00939 |
| NK4A214_group | Lachnoclostridium | -0.65486 | 0.000382 |
| Acetitomaculum | Roseburia | -0.65492 | 0.008989 |
| Olsenella | Turicibacter | -0.65592 | 0.000371 |
| Enterococcus | Lachnospiraceae_NK4A136_group | -0.6562 | 0.000368 |
| Sutterella | Helicobacter | -0.65716 | 0.000358 |
| Dorea | Acetitomaculum | -0.65941 | 0.008508 |
| Erysipelotrichaceae_UCG-003 | Succiniclasticum | -0.66311 | 0.000303 |
| Bifidobacterium | [Eubacterium]_eligens_group | -0.6659 | 0.00028 |
| Sutterella | Roseburia | -0.66603 | 0.007513 |
| Megamonas | NK4A214_group | -0.66924 | 0.000254 |
| Shuttleworthia | Bifidobacterium | -0.67172 | 0.006682 |
| Collinsella | Prevotellaceae_UCG-003 | -0.67572 | 0.00021 |
| Prevotellaceae_UCG-003 | Agathobacter | -0.67617 | 0.005997 |
| Lactobacillus | Erysipelotrichaceae_UCG-002 | -0.67825 | 0.000194 |
| Coprococcus | Acetitomaculum | -0.67918 | 0.000189 |
| Acetitomaculum | Agathobacter | -0.67956 | 0.005561 |
| Olsenella | Monoglobus | -0.67997 | 0.000185 |
| Erysipelotrichaceae_UCG-002 | Romboutsia | -0.68019 | 0.00555 |
| Alloprevotella | Sutterella | -0.68027 | 0.000183 |
| Prevotellaceae_UCG-003 | Fusicatenibacter | -0.68068 | 0.005539 |
| Collinsella | Erysipelotrichaceae_UCG-002 | -0.68071 | 0.00018 |
| Succiniclasticum | Romboutsia | -0.68239 | 0.005358 |
| Agathobacter | Lachnospiraceae_NK3A20_group | -0.6832 | 0.000167 |
| Erysipelotrichaceae_UCG-002 | Megamonas | -0.68371 | 0.005217 |
| Olsenella | Lachnospiraceae_UCG-004 | -0.6853 | 0.000157 |
| Enterococcus | [Eubacterium]_eligens_group | -0.68606 | 0.000153 |
| Fusicatenibacter | Erysipelotrichaceae_UCG-002 | -0.68637 | 0.000152 |
| Succiniclasticum | [Eubacterium]_hallii_group | -0.68637 | 0.005004 |
| Megamonas | Succiniclasticum | -0.68646 | 0.000151 |
| Erysipelotrichaceae_UCG-002 | Agathobacter | -0.68789 | 0.004874 |
| Intestinibacter | Succiniclasticum | -0.68816 | 0.000143 |
| Blautia | Erysipelotrichaceae_UCG-002 | -0.68899 | 0.00014 |
| Bifidobacterium | Erysipelotrichaceae_UCG-002 | -0.68899 | 0.00014 |
| Lachnoclostridium | Erysipelotrichaceae_UCG-002 | -0.68899 | 0.00014 |
| Dialister | Butyricicoccus | -0.68923 | 0.000139 |
| Succiniclasticum | Collinsella | -0.68953 | 0.004846 |
| Blautia | Succiniclasticum | -0.6901 | 0.000135 |
| Bifidobacterium | Succiniclasticum | -0.6901 | 0.000135 |
| Lachnoclostridium | Succiniclasticum | -0.6901 | 0.000135 |
| Fusicatenibacter | Succiniclasticum | -0.69023 | 0.000134 |
| Acidaminococcus | Megamonas | -0.69068 | 0.004846 |
| Lactobacillus | Succiniclasticum | -0.69146 | 0.000129 |
| [Eubacterium]_hallii_group | Erysipelotrichaceae_UCG-002 | -0.69188 | 0.000128 |
| Intestinibacter | Erysipelotrichaceae_UCG-002 | -0.69256 | 0.004846 |
| Agathobacter | Succiniclasticum | -0.6945 | 0.000117 |
| Lachnospiraceae_NK3A20_group | Coprococcus | -0.69827 | 0.004294 |
| Dialister | Anaerostipes | -0.6986 | 0.000103 |
| Shuttleworthia | Streptococcus | -0.69889 | 0.004294 |
| Coprococcus | Prevotellaceae_UCG-004 | -0.69973 | 9.90E-05 |
| Prevotella | [Eubacterium]_hallii_group | -0.69988 | 9.85E-05 |
| Agathobacter | Sutterella | -0.70067 | 9.60E-05 |
| Lactobacillus | Acetitomaculum | -0.71114 | 6.75E-05 |
| [Eubacterium]_hallii_group | Acetitomaculum | -0.71408 | 6.10E-05 |
| Lactobacillus | Lachnospiraceae_NK3A20_group | -0.71506 | 5.90E-05 |
| Prevotella | Blautia | -0.71629 | 5.65E-05 |
| Fusicatenibacter | Acetitomaculum | -0.71819 | 5.28E-05 |
| Olsenella | Romboutsia | -0.71877 | 0.002886 |
| Bacteroides | Erysipelotrichaceae_UCG-003 | -0.72374 | 4.33E-05 |
| Blautia | Dialister | -0.72385 | 4.32E-05 |
| Acetitomaculum | Collinsella | -0.73555 | 0.001914 |
| Romboutsia | Acetitomaculum | -0.73601 | 2.75E-05 |
| Lactobacillus | Olsenella | -0.73871 | 2.48E-05 |
| Agathobacter | Olsenella | -0.74703 | 1.79E-05 |
| Lactobacillus | Prevotellaceae_UCG-003 | -0.75419 | 1.33E-05 |
| Roseburia | Olsenella | -0.76955 | 6.90E-06 |
| Prevotellaceae_UCG-003 | Coprococcus | -0.77407 | 0.000743 |
| Acinetobacter | Intestinibacter | -0.80858 | 1.00E-06 |

| Supplementary Table S4: Co-occurrence network of genus bacteria in PCD patients | | | |
| --- | --- | --- | --- |
| genus1 | genus2 | r | p |
| Erysipelotrichaceae_UCG-002 | CAG-352 | 1 | 0 |
| Erysipelotrichaceae_UCG-002 | Succiniclasticum | 1 | 0 |
| Erysipelotrichaceae_UCG-002 | Prevotellaceae_UCG-004 | 1 | 0 |
| Luteimonas | Thermobacillus | 1 | 0 |
| Luteimonas | Cycloclasticus | 1 | 0 |
| Luteimonas | Methylotenera | 1 | 0 |
| Thermobacillus | Cycloclasticus | 1 | 0 |
| Thermobacillus | Methylotenera | 1 | 0 |
| Acetitomaculum | Prevotellaceae_UCG-003 | 1 | 0 |
| Cycloclasticus | Methylotenera | 1 | 0 |
| CAG-352 | Succiniclasticum | 1 | 0 |
| CAG-352 | Prevotellaceae_UCG-004 | 1 | 0 |
| Succiniclasticum | Prevotellaceae_UCG-004 | 1 | 0 |
| UCG-002 | Rikenellaceae_RC9_gut_group | 0.877373 | 3.89E-08 |
| Shuttleworthia | Family_XIII_AD3011_group | 0.866979 | 2.70E-05 |
| Alistipes | Barnesiella | 0.862794 | 1.18E-07 |
| Alloprevotella | Rikenellaceae_RC9_gut_group | 0.852558 | 2.40E-07 |
| Alloprevotella | UCG-002 | 0.851497 | 6.66E-05 |
| Dubosiella | Helicobacter | 0.843649 | 4.26E-07 |
| Lachnoclostridium | [Eubacterium]_hallii_group | 0.841897 | 9.81E-05 |
| Subdoligranulum | Dorea | 0.839788 | 5.41E-07 |
| Prevotellaceae_UCG-001 | Alloprevotella | 0.838145 | 9.81E-05 |
| Erysipelotrichaceae_UCG-002 | Acetitomaculum | 0.835109 | 7.16E-07 |
| Erysipelotrichaceae_UCG-002 | Lachnospiraceae_NK3A20_group | 0.835109 | 7.16E-07 |
| Erysipelotrichaceae_UCG-002 | Prevotellaceae_UCG-003 | 0.835109 | 7.16E-07 |
| Acetitomaculum | CAG-352 | 0.835109 | 7.16E-07 |
| Acetitomaculum | Succiniclasticum | 0.835109 | 7.16E-07 |
| Acetitomaculum | Prevotellaceae_UCG-004 | 0.835109 | 7.16E-07 |
| Lachnospiraceae_NK3A20_group | CAG-352 | 0.835109 | 7.16E-07 |
| Lachnospiraceae_NK3A20_group | Succiniclasticum | 0.835109 | 7.16E-07 |
| Lachnospiraceae_NK3A20_group | Prevotellaceae_UCG-004 | 0.835109 | 7.16E-07 |
| Prevotellaceae_UCG-003 | CAG-352 | 0.835109 | 9.81E-05 |
| Prevotellaceae_UCG-003 | Succiniclasticum | 0.835109 | 9.81E-05 |
| Prevotellaceae_UCG-003 | Prevotellaceae_UCG-004 | 0.835109 | 7.16E-07 |
| Ruminofilibacter | Amphibacillus | 0.832523 | 8.33E-07 |
| Family_XIII_AD3011_group | NK4A214_group | 0.832006 | 0.000111 |
| Family_XIII_AD3011_group | Holdemania | 0.827757 | 1.09E-06 |
| [Eubacterium]_hallii_group | Fusicatenibacter | 0.824809 | 0.000158 |
| Ruminococcus | Christensenellaceae_R-7_group | 0.821085 | 1.58E-06 |
| Blautia | Lachnoclostridium | 0.807312 | 3.23E-06 |
| UCG-005 | Rikenellaceae_RC9_gut_group | 0.793075 | 0.000724 |
| Cyanobium_PCC-6307 | hgcI_clade | 0.789043 | 7.66E-06 |
| Tyzzerella | Anaerostipes | 0.78734 | 0.000875 |
| Ruminofilibacter | Caldicoprobacter | 0.774977 | 1.41E-05 |
| Shuttleworthia | NK4A214_group | 0.772065 | 0.001553 |
| Blautia | Tyzzerella | 0.772008 | 1.60E-05 |
| Collinsella | Subdoligranulum | 0.77193 | 0.001553 |
| Romboutsia | [Eubacterium]_hallii_group | 0.762846 | 2.31E-05 |
| Holdemania | UBA1819 | 0.761126 | 0.002294 |
| Enterococcus | Streptococcus | 0.760703 | 2.51E-05 |
| Blautia | Bifidobacterium | 0.760069 | 2.58E-05 |
| Dubosiella | Faecalibaculum | 0.757508 | 0.002452 |
| Enterococcus | Klebsiella | 0.756249 | 2.98E-05 |
| Porphyromonas | Acetitomaculum | 0.754793 | 3.15E-05 |
| Porphyromonas | Prevotellaceae_UCG-003 | 0.754793 | 3.15E-05 |
| Lachnospiraceae_UCG-004 | Sarcina | 0.754282 | 3.22E-05 |
| Enterococcus | Lactococcus | 0.751678 | 3.55E-05 |
| [Eubacterium]_hallii_group | Intestinibacter | 0.75068 | 3.68E-05 |
| Anaerostipes | Blautia | 0.745059 | 0.003457 |
| Klebsiella | Haemophilus | 0.73571 | 6.32E-05 |
| Fastidiosipila | Ruminofilibacter | 0.734471 | 0.004822 |
| Romboutsia | Lachnoclostridium | 0.73419 | 6.66E-05 |
| Lachnoclostridium | Fusicatenibacter | 0.733877 | 0.004822 |
| Faecalibacterium | Subdoligranulum | 0.732213 | 7.13E-05 |
| [Ruminococcus]_torques_group | Blautia | 0.725476 | 0.006222 |
| Haemophilus | Intestinibacter | 0.723584 | 9.53E-05 |
| Lachnoclostridium | [Ruminococcus]_torques_group | 0.721028 | 0.00688 |
| Lachnoclostridium | Tyzzerella | 0.720574 | 0.000105 |
| Enterococcus | Cyanobium_PCC-6307 | 0.720435 | 0.000106 |
| NK4A214_group | Holdemania | 0.720247 | 0.000106 |
| Fastidiosipila | Amphibacillus | 0.718713 | 0.007052 |
| Ruminofilibacter | Turicibacter | 0.718643 | 0.000112 |
| Coprococcus | Klebsiella | 0.715098 | 0.007798 |
| Enterococcus | Haemophilus | 0.712091 | 0.000138 |
| Romboutsia | [Ruminococcus]_torques_group | 0.711144 | 0.000142 |
| Fastidiosipila | Proteiniphilum | 0.711107 | 0.008323 |
| Prevotella | NK4A214_group | 0.711001 | 0.000143 |
| Faecalibacterium | Monoglobus | 0.707221 | 0.000161 |
| [Eubacterium]_hallii_group | [Ruminococcus]_torques_group | 0.707191 | 0.009144 |
| Erysipelotrichaceae_UCG-003 | Fusicatenibacter | 0.700618 | 0.010909 |
| Alloprevotella | UCG-005 | 0.700124 | 0.0002 |
| [Eubacterium]_hallii_group | Blautia | 0.698617 | 0.011314 |
| Collinsella | Dorea | 0.697598 | 0.000215 |
| Intestinibacter | Romboutsia | 0.693847 | 0.012733 |
| Lachnoclostridium | Butyricicoccus | 0.690559 | 0.000265 |
| Erysipelotrichaceae_UCG-003 | Faecalibacterium | 0.690064 | 0.0139 |
| Butyricicoccus | Tyzzerella | 0.683573 | 0.000323 |
| Lachnospiraceae_NK3A20_group | Acetitomaculum | 0.682277 | 0.016492 |
| Lachnospiraceae_NK3A20_group | Prevotellaceae_UCG-003 | 0.682277 | 0.000335 |
| Intestinibacter | Fusicatenibacter | 0.682155 | 0.016492 |
| Ruminococcus | Parabacteroides | 0.678695 | 0.017971 |
| [Eubacterium]_hallii_group | Tyzzerella | 0.676064 | 0.000398 |
| Holdemania | Shuttleworthia | 0.675123 | 0.019421 |
| Paraprevotella | Prevotellaceae_UCG-001 | 0.673509 | 0.000427 |
| Cyanobium_PCC-6307 | unidentified_Chloroplast | 0.67158 | 0.00045 |
| Lactococcus | Klebsiella | 0.671135 | 0.020587 |
| Enterococcus | hgcI_clade | 0.668021 | 0.000495 |
| Parabacteroides | Christensenellaceae_R-7_group | 0.66774 | 0.000499 |
| Butyricicoccus | [Eubacterium]_hallii_group | 0.667326 | 0.02194 |
| Lachnoclostridium | Intestinibacter | 0.666667 | 0.000513 |
| Enterococcus | Citrobacter | 0.66106 | 0.000594 |
| Blautia | Romboutsia | 0.659091 | 0.000625 |
| Lachnospiraceae_NK4A136_group | Alloprevotella | 0.657542 | 0.027047 |
| Romboutsia | Tyzzerella | 0.656281 | 0.000672 |
| Turicibacter | Caldicoprobacter | 0.654815 | 0.028484 |
| [Ruminococcus]_gnavus_group | Enterococcus | 0.650669 | 0.031072 |
| Turicibacter | Amphibacillus | 0.649424 | 0.000799 |
| Barnesiella | Holdemanella | 0.648031 | 0.032509 |
| Megamonas | Dorea | 0.647823 | 0.000831 |
| Olsenella | Acetitomaculum | 0.647012 | 0.032618 |
| Olsenella | Prevotellaceae_UCG-003 | 0.647012 | 0.000848 |
| UCG-005 | UCG-002 | 0.642317 | 0.036287 |
| Subdoligranulum | Monoglobus | 0.64095 | 0.000983 |
| Prevotellaceae_UCG-001 | UCG-002 | 0.639372 | 0.038159 |
| Megamonas | Butyricicoccus | 0.63915 | 0.001026 |
| UBA1819 | [Ruminococcus]_torques_group | 0.638893 | 0.038159 |
| Megasphaera | Proteiniphilum | 0.637599 | 0.001065 |
| Lachnospiraceae_NK3A20_group | Acinetobacter | 0.636641 | 0.039638 |
| [Ruminococcus]_gnavus_group | Haemophilus | 0.635825 | 0.001111 |
| Paraprevotella | UCG-002 | 0.630967 | 0.044609 |
| [Ruminococcus]_gnavus_group | Escherichia-Shigella | 0.628458 | 0.046544 |
| Megasphaera | Fastidiosipila | 0.628156 | 0.00133 |
| Enterococcus | Intestinibacter | 0.627073 | 0.001363 |
| Neisseria | Acetitomaculum | 0.625811 | 0.001403 |
| Neisseria | Prevotellaceae_UCG-003 | 0.625811 | 0.001403 |
| Holdemanella | Lactococcus | 0.623536 | 0.001478 |
| Klebsiella | Cyanobium_PCC-6307 | 0.62284 | 0.001502 |
| Agathobacter | Roseburia | 0.62253 | 0.001512 |
| Turicibacter | Helicobacter | 0.622417 | 0.001516 |
| Ruminofilibacter | Proteiniphilum | 0.621409 | 0.001551 |
| unidentified_Chloroplast | hgcI_clade | 0.615833 | 0.001757 |
| Alloprevotella | Dubosiella | 0.613688 | 0.001842 |
| Parasutterella | Lachnospiraceae_NK4A136_group | 0.613597 | 0.001846 |
| Caldicoprobacter | Fastidiosipila | 0.613169 | 0.001864 |
| Caldicoprobacter | Amphibacillus | 0.611982 | 0.001913 |
| Raoultella | Citrobacter | 0.611499 | 0.001933 |
| Faecalibacterium | Fusicatenibacter | 0.611317 | 0.001941 |
| Lachnospiraceae_NK4A136_group | Rikenellaceae_RC9_gut_group | 0.609657 | 0.002012 |
| Dialister | UCG-005 | 0.609008 | 0.002041 |
| [Ruminococcus]_gnavus_group | Intestinibacter | 0.608352 | 0.00207 |
| Klebsiella | Dubosiella | 0.607906 | 0.00209 |
| Catenibacterium | Acidaminococcus | 0.606951 | 0.002134 |
| Paraprevotella | Alloprevotella | 0.605869 | 0.002184 |
| Akkermansia | Family_XIII_AD3011_group | 0.603533 | 0.002296 |
| Blautia | Erysipelotrichaceae_UCG-003 | 0.602571 | 0.002343 |
| Family_XIII_AD3011_group | Catenibacterium | 0.601702 | 0.002387 |
| Romboutsia | Fusicatenibacter | 0.601433 | 0.0024 |
| Intestinibacter | Tyzzerella | 0.601039 | 0.00242 |
| Phascolarctobacterium | Catenibacterium | -0.60223 | 0.00236 |
| Prevotella | Anaerostipes | -0.60474 | 0.002237 |
| [Ruminococcus]_gnavus_group | Parabacteroides | -0.60751 | 0.002108 |
| Roseburia | Paraprevotella | -0.6088 | 0.00205 |
| Dialister | Collinsella | -0.609 | 0.002041 |
| Megamonas | Lachnospira | -0.60983 | 0.002005 |
| Megamonas | Acinetobacter | -0.61135 | 0.00194 |
| Phascolarctobacterium | Prevotella | -0.62942 | 0.045892 |
| Fusicatenibacter | Sutterella | -0.65151 | 0.000758 |
| Dorea | Acinetobacter | -0.66191 | 0.024828 |
| Parasutterella | Clostridium_sensu_stricto_1 | -0.67029 | 0.000466 |
| Acidaminococcus | Monoglobus | -0.67118 | 0.020587 |
| UCG-002 | Tyzzerella | -0.67128 | 0.000454 |
| Dorea | Dialister | -0.68247 | 0.016492 |
| Megamonas | Dialister | -0.7067 | 0.000164 |
| Acinetobacter | Subdoligranulum | -0.71246 | 0.008323 |
| Anaerostipes | UCG-002 | -0.75809 | 2.78E-05 |
| Acinetobacter | Collinsella | -0.78821 | 0.000861 |
